# A digital heat early warning system for older adults

**DOI:** 10.1038/s41746-025-01505-5

**Published:** 2025-02-20

**Authors:** Mehak Oberai, Zhiwei Xu, Aaron Bach, Connor Forbes, Ella Jackman, Fergus O’Connor, Isabella Ennever, Sebastian Binnewies, Steven Baker, Shannon Rutherford

**Affiliations:** 1 School of Medicine and Dentistry, Griffith University, Gold Coast, Queensland Australia; 2 Cities Research Institute, Griffith University, Gold Coast, Queensland Australia; 3 School of Health Sciences and Social Work, Griffith University, Gold Coast, Queensland Australia; 4 School of Information and Communication Technology, Griffith University, Gold Coast, Queensland Australia; 5 School of Pharmacy and Medicine, Griffith University, Gold Coast, Queensland Australia

**Keywords:** Natural hazards, Developing world

## Abstract

Extreme heat events lead to considerable health burden and are becoming more severe and frequent, calling for the development of effective population-based and individualised heat early warning systems. We developed an individualised heat early warning system and tested it in 78 older adults’ ( ≥ 65 years) homes in Southeast Queensland, Australia. Quantitative and qualitative data from this proof-of-concept testing study showed that the Ethos system performed well on a standard usability scale (mean score of 78 on the System Usability Scale). Following a summer-time use of this early warning system, there were increases in heat preparedness (*P* < 0.001, marginal homogeneity tests) but no significant increases in heat health risk perception or the uptake of low-cost cooling measures (e.g., hand/forearm bath, fans). This proof-of-concept research demonstrated the usability of this tailored, actionable, real-time digital heat early warning system, although the effectiveness of the system remains to be evaluated in a robust trial design.

## Introduction

Anthropogenically-driven climate change continues to lead to multifaceted and widespread effects on human health, particularly through extreme heat events (i.e., short-term periods [<3 days] of significantly higher than average temperatures) and heatwaves (i.e., prolonged periods [≥3 days] of abnormally high temperatures)^1,2^. At an individual level, the high ambient temperatures associated with periods of extreme heat place significant strain on the cardiovascular, respiratory, renal, and nervous systems, increasing the likelihood of heat-related illnesses (e.g., heat exhaustion) and an exacerbation of pre-existing chronic diseases (e.g., cardiovascular diseases)^3^, leading to increased population-wide demand on healthcare services^3,4^. Older adults ( ≥ 65 years) are at greater risk of adverse health outcomes during periods of extreme heat due to age-related impairments in thermoregulatory function^5–8^, a greater prevalence of chronic diseases^9^, increased use of prescription medications that may hinder heat loss capacity (e.g., anti-adrenergics, beta-blockers, diuretics, anticholinergics, antipsychotics)^9^, and higher prevalence of financial, social, and environmental inequalities^6,10^.

Higher risk perception is known to drive behaviour change and increase risk preparedness and resilience in the population^11^. Although older adults are at greater risk of heat-related health effects, studies to date have shown that they have poor heat health risk perception^12–15^. Effective behavioural responses to heat must be preceded by a perceived need to act, which can be influenced by risk perception, as well as interactions between physiological status and perception^6^. While older adults remain capable of initiating appropriate behavioural responses during acute heat stress, these may occur at heightened levels of thermal and cardiovascular strain compared to their younger counterparts^16,17^. This may inadvertently exacerbate their already heightened risk during heat exposure. Considering a general desire to age in place^18^, in conjunction with the fact that older adults spend as much as 90% of their time indoors^19^, and most heat-related fatalities occur within the home^20^, there is a clear need for effective heat management strategies for reducing heat stress in the home for this age group. In this context, indoor temperatures have been shown to be strongly related to outdoor temperature, particularly during periods of hot weather^21^, thus highlighting potential vulnerability within the home. In Queensland, Australia, it was estimated that ~84% of households have access to air-conditioning to provide in-home space cooling during periods of hot weather^22^. While air-conditioning is extremely effective at lowering the ambient temperature of the indoor microclimate, and therefore effectively eliminating the dangers of heat exposure, older adults may be less likely to utilise air-conditioning during periods of hot weather due to the high financial cost of operation^23^.

A critical component of heat-health action plans, which are designed to reduce the population-wide burden from the increasing frequency, duration, and intensity of periods of extreme heat, is the effective implementation of heat early warning systems^1^. Heat early warning systems aim to enhance heat preparedness^24^ by utilising meteorological forecasting and related methods to communicate alerts to initiate population-wide interventions that mitigate the impact of heat stress on human health during periods of extreme heat. While the Intergovernmental Panel on Climate Change (IPCC), World Meteorological Organization (WMO), and World Health Organization (WHO), are in agreeance that heat early warning systems reduce the risk of heat-related deaths^25^, and epidemiological data suggest that heat early warning systems save lives^26^, there are inherent limitations associated with population-based heat early warning systems. For example, existing population-based heat early warning systems rely on outdoor weather data as a proxy for indoor exposure^10^ and may not effectively account for individual factors that influence susceptibility to environmental heat exposure. As such, they often fail to correctly estimate and notify older adults of their heat health risk and guide them to take suitable behavioural action^5,15,27–29^, particularly during periods of hot weather (occurring outside of heatwaves). For example, while the relation between heatwaves and mortality risk is clear, epidemiological evidence suggests that hot weather per se (i.e., not occurring in heatwaves) also increases population-wide mortality^30^. Further, population-based heat early warning systems lack individualised cooling suggestions^10^, assuming that broad recommendations communicated to the population are accessible and acceptable to those most at risk.

Increasing advancements in the usability, ubiquity and utilisation of information and communication technology have contributed to its increasing use in supporting climate change adaptation and mitigation^31,32^. This has coincided with the increasing technology literacy and utilisation of information and communication technology by older adults^33^, especially those seeking to age in place. However, there is currently a lack of individualised heat early warning systems designed for older adults despite the ability of such systems to overcome the limitations of existing population-based heat early warning systems. As a result, there are increasing calls for the development of individualised heat early warning systems^1,34,35^.

To address this critical knowledge gap, we developed the Ethos (**E**x**t**reme **h**eat and **o**lder person**s**) system—an in-home monitoring device that acts as an individualised early warning system that provides tailored heat management suggestions for older adults to improve heat resilience. The Ethos system utilises direct environmental monitoring within an individual’s home, while accounting for individual factors that influence heat susceptibility to generate individualised heat alert warnings and cooling recommendations. It was piloted from December 2023 to February 2024 (Australian summer), with older adults residing in subtropical Queensland, Australia.

Via this 3-month study, we investigated the potential efficacy of the Ethos system (the intervention) as a decision support and early warning household tool to improve heat preparedness and resilience among older adults during hot weather. The Ethos system aims to do this by advancing awareness and knowledge about heat-related health risks, improving risk perception through alerting, and encouraging better heat management through enhanced response capacity drawing on the four elements of people-centred early warning systems^36^. This paper presents findings based on quantitative and qualitative data collected on key issues of heat health risk awareness, risk perception, and cooling actions. The usability and acceptability of the intervention were also investigated.

## Results

### Sample size & characteristics

88 older adults ( ≥ 65 years) were recruited across Southeast Queensland, Australia, using defined recruitment criteria (details in the methods section). Data from five persons were excluded from analysis because of a-prior engagement with the project (which could have biased their results), a further three people withdrew during the data collection period (health and family issues), and two participants did not complete the survey during the post-intervention stage. Hence, data from 78 participants was included in the final analysis. Their socio-demographic and dwelling characteristics are presented in Table 1. The participants were mainly from three cities (Supplementary Fig. 1): Brisbane, Gold Coast, and Sunshine Coast.

**Table 1:** Socio-demographic and housing characteristics of the participants

Demographics	Description	*n* (%)
Age (years (yr))	65–69	17 (22)
	70–74	22 (28)
	75–79	19 (24)
	80+	20 (26)
Gender	Males	37 (47)
	Females	41 (53)
Education	Diploma or higher education	52 (67)
	Secondary school or lower	26 (33)
Personal weekly income (USD)	<450	46 (58)
	>450	32 (42)
Household weekly income (USD)	<835	62 (80)
	>835	16 (20)
Living situation	Living alone	32 (41)
	Living with partner only	33 (42)
	Living with family/friends	6 (8)
	Other	7 (9)
Housing characteristics		
Type of housing	Unit or apartment or flat	7 (9)
	Townhouse/Villa	6 (8)
	Duplex/dual occupancy/cluster house/seniors’ unit	10 (12)
	Free standing house	53 (68)
	Other	2 (3)
Housing material	Brick	48 (61)
	Concrete	10 (13)
	Timber	10 (13)
	Other	10 (13)
Number of storeys	Single storey	58 (74)
	Double storey	6 (18)
	Multiple storey	14 (8)
Number of bedrooms	1	5 (6)
	2	13 (17)
	3	32 (41)
	4	24 (31)
	5 or more	4 (5)
Tenancy	Ownership	60 (77)
	Renting - private	11 (14)
	Renting - community housing/public housing	3 (4)
	Other	4 (5)
Home insulation	Yes	57 (73)
	No	13 (17)
	Don’t know	8 (10)
Home air-conditioning	Yes	70 (90)
	No	8 (10)

The participants had an average age of 75 years (range: 65–100, standard deviation: 7). Among the 78 participants, 70 (percentage: 90%) had air-conditioning at home and 53 (68%) lived in free-standing houses. Thirty-one participants (41%) lived alone and 33 lived (42%) with their partner. The participants had an average self-reported quality of life (measured by EQ-D5-5L) score of 0.90 (range: 0.43–1.0). Forty-one participants (53%) had self-reported cardiac disorders, 20 (26%) had self-reported endocrine disorders, and 17 (22%) had self-reported respiratory disorders.

### Technology acceptance

The Senior Technology Acceptance Model (STAM) questionnaire included in the survey of the pre-intervention stage indicated a mean score for technology acceptance of 98 (range 27–124, standard deviation:19), suggesting that our sample had an average positive attitude (21.6 for attitudinal beliefs and 28.4 for control beliefs) towards using technology with limited gerontechnology anxiety (7.7). The mean STAM score remained almost the same in the post-intervention stage (mean: 97, standard deviation: 19), although the score range was wider (range: 16-135).

### Ethos system alerts and participants’ self-reported cooling behaviours during the intervention stage

**Fig. 1:**
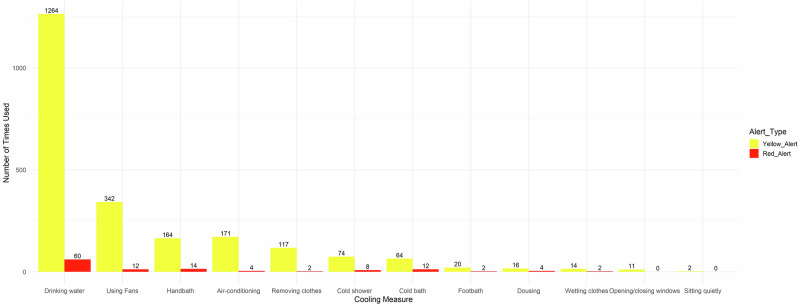
The total number of cooling measures adopted in response to alerts issued by the Ethos system, from 1st December 2023 to 29th February 2024. Data was recorded on the evening following an Ethos system issued alert warning. One participant could report using multiple cooling measures following one alert.

Over the three-month intervention stage, a total of 2346 yellow alerts and 88 red alerts were issued across the 78 households. The participants were home when 58% (1351/2346) yellow and 75% (66/88) red alerts were issued, respectively. Participants reported that, following 90% (1221/1351) of the yellow alerts and 94% (62/66) of the red alerts, they used cooling strategies recommended by the Ethos system. The top 5 cooling strategies they used after hearing the alerts included drinking more water, turning on the fan, hand bath, turning on air-conditioning, and removing clothing or wearing lighter clothes (Fig. 1).

### Improved heat preparedness among the participants

Quantitative analysis of data collected in the pre- and post-intervention surveys suggest that having the Ethos system at a participant’s residence was associated with improved heat preparedness. Compared with the pre-intervention stage, a higher proportion of older adults felt that they were well prepared for heatwaves in the post-intervention stage (percentage 69% [number of participants 54] vs. 85% [66]; *p* < 0.001, marginal homogeneity test). Moreover, the percentage of participants who reported feeling uncertain about preparedness for heatwaves decreased from 19% in the pre-intervention stage to 0% in the post-intervention stage (*p* < 0.001, marginal homogeneity test). Heat preparedness increased by 3.4 times among the participants from the pre-intervention stage to the post-intervention stage (*p* < 0.001, marginal homogeneity test).

Qualitative analysis results provide further insights into heat preparedness (Supplementary Table 1) ranging from simple statements regarding improved personal preparedness, *“better prepared to handle the hot conditions*” (Clara,70 yr), to more elaborated considerations for planning and blending personal and household cooling strategies, “*If temperature is less than 30* *°C relax. If 30 - 33* *°C open up doors windows etc and use a fan, shed clothes. If greater than 33* *°C shut up house, set air conditioner to 27* *°C and turn on”* (Alvin, 81 yr). In addition, findings demonstrated the empowerment of participants to make data-informed decisions about daily household modifications, “*When the temperature outside began to rise, I would shut doors and windows…I also used the yellow warnings as prompts to begin cooling strategies e.g., remove excess clothing”* (Nick, 73 yr). Participant feedback demonstrated that the system also boosted their confidence in taking adaptive action including knowing what actions to take in the future.

Participants also indicated future changes in terms of preparing for hot days as a result of participating in the trial, *“far better understanding of heat and the impact it has and what I need to do in the future”* (Lara, 75 yr).

### Enhanced awareness and perception about heat health risks in the participants

In the pre-intervention survey, all participants reported that they had heard heatwave warnings issued by the Australian Bureau of Meteorology. Compared to pre-intervention testing, the participants were more informed about heatwaves and associated health consequences post the intervention testing (13% [10] vs. 36% [28], *p* < 0.001, marginal homogeneity test). 13% [10] reported they were extremely concerned about the effects of heatwaves on themselves personally at the end of the intervention testing compared to 1% [1] at the start (*p* = 0.201, marginal homogeneity test). A total of 47% [36] of the respondents strongly agreed that heatwaves pose a risk to their health post- the intervention testing compared to 32% [25] at the pre-intervention stage (*p* = 0.09, marginal homogeneity test). Compared to the beginning of the study, participants were 4.3 times more likely to be well informed about heatwaves and its consequences to their health compared to at the end of the testing period (*p* < 0.001; marginal homogeneity test).

Qualitative findings (Supplementary Table 2) further support these quantitative findings. The use of the Ethos system was related to participants being more aware and having better knowledge about heat and related health risks particularly as an older adult. *[It] “Made me more aware of the effects of heat on older persons like myself*” (Harvey, 69 yr). A common theme expressed by the participants was awareness of the way temperature and humidity interact with each other making the environmental conditions worse on a hot day, *“it raised awareness of the confluence between temperature and humidity, and, in what situations, these two factors lead to taking specific actions*” (Russell, 73 yr).

Qualitative findings also highlight the value of the system to increase spatial and temporal knowledge of temperature and humidity within the participant’s household and surroundings, *“I was more conscious of the temperature in my home and how to adjust my day accordingly*.” (Dorothy, 71 yr), and *“I know which areas in the house are cooler and where to avoid when it gets hot*” (Della, 75 yr).

### Better heat management through increased response capacity

**Fig. 2:**
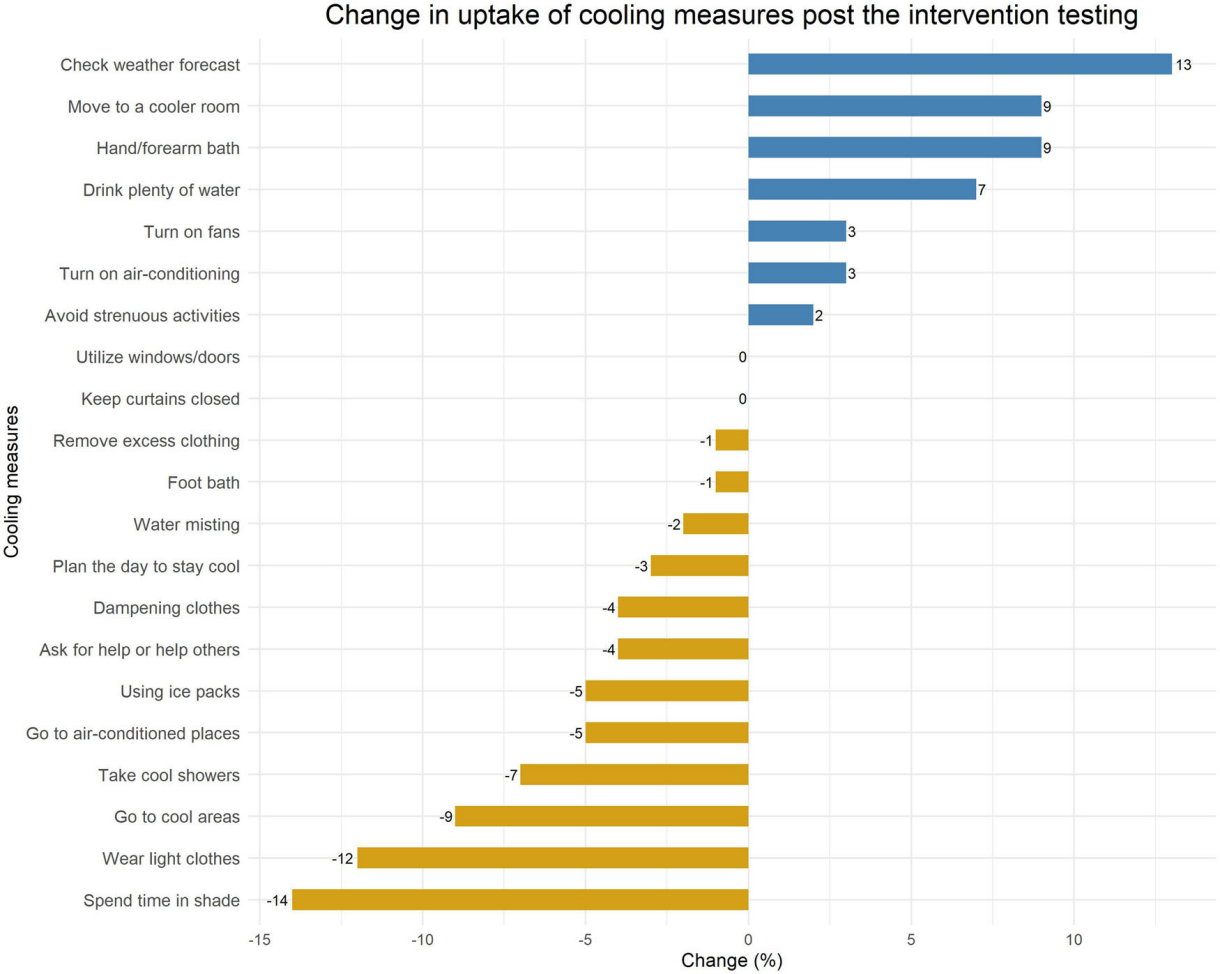
Changes in the uptake of cooling behaviours from pre-intervention to the post-intervention stage. Negative values indicate decreases in cooling behaviour use from the pre-intervention to post-intervention stage. Positive values mean increases in cooling behaviour use.

Changes in the use of diverse cooling measures pre- and post-intervention testing were observed. Figure 2 shows the measures for which frequency of usage changed post intervention. The uses of cooling measures which changed the most were checking weather forecast (from 67% [number of participants reporting using the cooling measure: 52] to 80% [62]), hand/forearm bath (from 10% [8] to 19% [15]), moving to a cooler room in the house (from 63% [49] to 72% [56]) and drinking plenty of water (from 87% [68] to 94% [73]). There were decreases in the use of some cooling measures, including spending time in shade (from 87% [68] to 73% [57]), wearing loose fitting clothes (from 86% [67] to 74% [58]), going to cooler outdoor places (from 13% [10] to 4% [3]) and taking cool showers or splashing with water (from 47% [37] to 40% [31]). Despite the changes in the use of cooling measures from the pre-intervention stage to the post-intervention stage, none of the changes were statistically significant (*p* > 0.05, McNemar test).

Qualitative findings provide further insights into use of cooling measures including air-conditioning, combination of cooling measures, timing of cooling and other protective behaviours, and cooling option diversity awareness (Supplementary Table 3). Participants reported being more strategic, *“More use of the air-conditioner. I also closed off the hottest rooms from the rest of the house*” (Dorothy, 71 yr), and more confident (feelings of reassurance about need to take action) in when and how they used air-conditioning, *“I had moral support when I needed to turn on the A/C and close up”* (Emma, 100 yr).

While there was no significant change in the use of cooling interventions following recommendations made by the Ethos system, some individuals reported using fans more, and in conjunction with air-conditioning, “*I found a combination approach useful. Fan and air-conditioning together with air-conditioning at 26* *°C worked well & saved money*” (Callum, 81 yr). Some participants reported that the Ethos system improved their knowledge of more diverse cooling options such as the use of blinds, delaying physical activities until the end of the day and using forearm baths.

### Usability and acceptability of the Ethos system

**Fig. 3:**
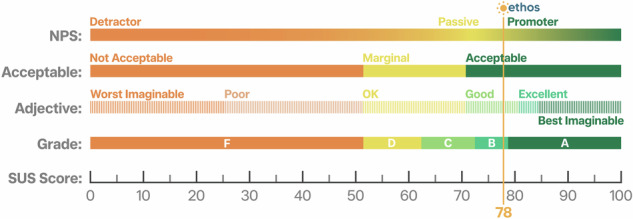
Interpretation of the usability score of the Ethos system (Figure adapted from Sauro, 2018^39^). System usability scale (SUS) is the most widely used standardised questionnaire for the assessment of perceived usability^81^ and has been utilised in the past for testing the usability of various commonly used digital interfaces and devices such as an iPhone, or an application like Amazon^82^. It consists of 10 questions and can be positively worded or can have a mix of both positively worded and negatively worded statements^81^. The scores are predicted as adjectives as shown in this Fig. 3. We used a positively worded SUS scale to test the usability and acceptance of the Ethos system. Based on the SUS results the Ethos system was acceptable and rated as good by the users.

The mean score of the Ethos system usability, measured by the System Usability Scale (SUS), was 78 (standard deviation: 18, range: 47.5–100). As depicted in Fig. 3, this usability score indicates the Ethos system to be acceptable^37^. The usability of the Ethos system was between good and excellent on the adjective scale of usability^38^ with the net promoter rating (NPS) between the boundary of being passive to promoter^39^. This promoter rating aligns with qualitative post-intervention comments—made by some participants reporting that they shared the information provided by the Ethos system to friends and family, *“Shared with friends the info of temperature and ways to cool down”* (Hazel, 79 yr).

Qualitative findings about the Ethos system’s usability and its impact on the participants provide a deeper understanding of its usability (Supplementary Table 4). Common themes of usability related to the interface design *“I liked the size and the design. It was clear and the colours easily seen from a distance* (Emma, 100 yr)” and its alerting value “*The green and yellow (we never got a red!!) was easily noticeable even at a quick glance* (Russell,73 yr)*”*, the simplicity and ease of use: ”*I liked the whole package: neat, easy to read and operate* (Esther, 86 yr)*.”*, and the value of the spatial real-time information it provided*, “I liked knowing the temperature and humidity levels in the different areas of my home. That way, I moved to the coolest area, or, turned on air conditioning, whichever was better at the time* (Daisy, 76 yr)*”*.

## Discussion

This paper presents the main findings of the in-home proof-of-concept testing of an individualised heat early warning system (i.e., the Ethos system) in a sample of older persons in subtropical Southeast Queensland, Australia. Our findings provide key insights into the value of digital interventions to improve heat awareness and heat health risk perception and influence personal and household cooling practices. The findings suggest that using the Ethos system during the summer period was associated with higher heat preparedness among the participants. Notably, greater than >90% of alert warnings issued by the Ethos system were followed by self-reported behavioural change. Overall, participants reported being more spatially and temporally aware of the temperature and humidity within their home and their ability to better relate the temperature and humidity within their homes with their perception of heat. There was an uptake of personal heat action plans in preparation for future heat events. These findings are consistent with the role that early warning systems play in early stages of disaster management by encouraging efficient short-term preparation and response^40^.

A critical component of heat preparedness is timeliness and willingness to act when exposed to environmental extremes^1^. Perhaps the most significant finding resulting from our proof-of-concept testing study is that 90% of yellow alerts and 94% of red alerts issued by the intervention were reported to be acted upon by participants. This aligns with key features of an early warning system which are to ensure people who are at risk are able to receive the alert (detection and communication), understand it (risk knowledge), and most importantly, act on it (response capacity)^40,41^. Despite no significant changes in the number and diversity of cooling strategies and behaviours utilised by participants, qualitative responses suggested that some participants modified their use of air-conditioning, fans, consumed more fluids, and monitored weather forecasts to prepare in advance of hot weather. These results are in accord with findings reported by others^12,35,42^.

Of particular interest, some participants reported strategically using air-conditioning with fans, thus potentially decreasing the reliance on carbon-intensive air-conditioning to provide thermal relief^43^. Similarly, participants reported modifying their household or retrofitting building level cooling interventions such as the installation of external blinds and shutters, suggesting the intervention also prompted household adaptations that would increase their ongoing household heat resilience. The intervention was designed as a decision support tool, with heat alerts translated to participants as ‘nudges’ where suggestions regarding behaviour change, rather than commands were issued. This was done to preserve the autonomy of the users. Notably, nudging involves designing choice architectures that encourage individuals to make decisions that align with their best interests and societal goals, without limiting personal freedom of choice^44,45^. In this context, it appears that nudging strategies during times of need (i.e., when predicted core body temperature is increasing) may have promoted more efficient uptake of personal cooling interventions. While some participants reported utilising air-conditioning in conjunction with fans, others reported utilising air-conditioning alone to a greater degree than they otherwise would have.

Thus, our proof-of-concept design displays promising utility as an individualised early warning system that can effectively translate risk, which may lead to behaviour change of participants. While it is not possible to make direct conclusions regarding risk reduction behaviour from this study design, the fact that participants reported acting upon heat-alert warnings issued by the intervention (and implementing subsequent behavioural changes) is highly encouraging. While cooling suggestions provided by the intervention were prioritised in order of claimed effectiveness^46^ as well as user preferences, future investigations should look to nuance messaging translated by nudging systems regarding the most effective and efficient utilisation of air-conditioning, amongst the suite of other cooling mechanisms recommended. Thus, not only providing protection for the most vulnerable but also assisting users to save on energy costs and be more prepared should energy-based cooling be unavailable to them (e.g. during power outages).

Another significant outcome was that participants found the intervention to be useful and acceptable to the extent that some expressed a wish to keep the system. The acceptable system usability score of 78 has also been associated with a verbal user rating of ‘*good’* in other studies^42,43^. Moreover, the usability score of the intervention is higher than commonly used digital devices such as Global Positioning System (GPS) (SUS-70.8) and Digital Video Recorder (DVR) (SUS-74.0), and is comparable to a reported usability score of an iPhone (78.5)^37^. Studies have shown that cooling behaviours in individuals aged over 65 years vary in their acceptability and accessibility among this population^2,15,34^. In the intervention tested, cooling suggestions were also prioritised by user preferences (based on user acceptability and accessibility) to incorporate their perspective into decision making and align with their personal values. Including the nudging along with these elements may have established trust between users and the intervention thereby, influencing the perception of system usability and acceptance of the system as a useful intervention^44^. In addition, the system design incorporated a co-design participatory approach^47^, where a project reference group consisting of older adults and carers was involved at various stages of system development to gather input on the system design and its features. This ensured the needs of end-users were considered and explored and this process is likely to have contributed to increased acceptability of the digital Ethos system among the participants, thereby, highlighting the importance of using participatory research, analogous to the findings of others^48–51^.

Arguably, the nudges provided (via displayed information and alerting) along with the simplicity of the design of the system were associated with increased knowledge and awareness of general and personal heat health risk and spatial and temporal home temperature and humidity, and diverse and effective personal and household cooling measures. Qualitative data suggests that this leads to more anticipatory cooling and a more strategic use of diverse cooling strategies that have the potential to reduce cost (e.g., by more efficient use of high-cost cooling like air-conditioning) and that provide resilience when active cooling requiring energy is not accessible (e.g., as a result of power outages associated with poor energy security or following other climate related extreme events such as bushfires or storms)^52^.

These early findings suggest this type of digital system has the potential to increase heat preparedness and resilience among the population, providing some evidence of the role of individualised decision support technologies in promoting climate adaptation and action. These findings are in accordance with previous literature^53–55^ where decision making tools have been shown to reduce the heat burden by supporting individual and population level decision making. Digital technologies are increasingly being used to promote healthy ageing and improve the well-being of older populations choosing to age in place. Fall-prevention and communication devices are some of the commonly used technologies to have have been tested and used to enhance ageing in place^56^. Similar digital technologies such as smart phone applications^57,58^, wearable technologies^59,60^, and online tools such as websites and programs^54,55^ have been developed to protect people from heatwaves but need more research and testing. Compared with the existing digital technologies aiming to protect people from heatwaves, the Ethos system is unique because it incorporates indoor environment measurement, prediction of individual heat health risk, and recommendations for efficacious, accessible, and acceptable cooling measures. Incorporating activity data (e.g., step counts) and physiological measurements (e.g., heart rate and heart rate variability) derived from wearable devices, in conjunction with environmental monitoring may provide greater insight as to overall risk, as well as the effectiveness of individualised early warning systems to reduce risk for vulnerable populations during exposure to indoor overheating^61^. This proof-of-concept trial of the Ethos system has demonstrated its potential to be an effective intervention for addressing the projected global heat and health impacts, particularly for the most vulnerable. By considering factors such as geographical location, household level environmental conditions, and various personal/individual health drivers that determine heat health risk, providing alerts when risk is increasing, considering increased digital literacy in older adults, increased rate of digitalisation, and facilitating and tailoring an individualised set of cooling recommendations, the Ethos system has the potential to overcome many of the limitations of existing early warning systems.

Despite these early positive signs of impact and usability of the intervention, various limitations require consideration. First, as this initial study period was designed as a proof-of-concept to assess the efficacy of the intervention as an individualised early warning system, we recruited a relatively small sample size (88 households) which are not entirely representative of the population. While the percentage of total participants in the current cohort that had home air-conditioning reflects the local state average^22^, we acknowledge that this cohort of individuals may not be entirely reflective of older adults in Queensland. Further, no objective information was recorded detailing explicit times when individuals chose to utilise air-conditioning or not, and thus beyond the results presented from the pre- to post-summer questionnaire, it remains unclear how and why individuals chose to use air-conditioning (or not).

Moreover, it was not possible to quantify radiant heat, or air movement within individuals’ homes, factors which both influence physiological heat exchange. Thus, there was an underlying assumption of minimal to no air movement and no direct sunlight exposure within the home, which may have misrepresented the occupant’s actual exposure to heat stress, which would in-turn influence the predictive capability of the JOS-3 model. To this point, while the JOS-3 model is a validated core temperature prediction model^62^ we acknowledge the limitations of utilising predictive equations to predict core temperature trajectories over the forthcoming 9 h to estimate exposure risk. Therefore, we emphasise that the Ethos system should be considered one component of comprehensive heat health monitoring, and not a definitive predictor of all heat-related health risks and that core temperature alone may not fully encapsulate the risk of adverse events. Future models could look to integrate other physiological markers (e.g., heart rate) to enhance predictive power.

In addition, it is important to consider that the intervention period coincided with El-Nino summer conditions^63^, which may have influenced the post-intervention survey results (as a result of heightened heat awareness through media exposure among other factors). Second, it was not possible to capture the average response time for completed surveys, which may have provided greater insights into user engagement with the Ethos system. Further, while individuals included within the intervention period reported being aware of the Ethos system alerts issued and subsequently modified behaviour as a result, we acknowledge that this proof-of-concept study was unable to objectively ascertain changes in behaviour which may reduce risk. Finally, surveys issued to participants were specifically designed to assess the application of the intervention, and thus have not been validated as gold-standard methods of measurement of effectiveness. Future studies should look to ascertain the efficacy of the system within more diverse population settings and climates as well as looking to obtain objective information regarding the appropriate implementation of risk-reducing behaviours.

The proof-of-concept testing of Ethos system, provides an example of an individualised heat early warning system with demonstrated early indication of its usability, acceptance, and effectiveness as a promising technology in enhancing heat resilience among older adults, promoting healthy ageing, and supporting efficacious cooling practices. This research highlights the value of co-designed technology and digital systems in mitigating the adverse health effects of climate change, such as increasing extreme temperatures, in specific, and large at-risk populations. This digital system represents a climate adaptation strategy for prompting at-risk populations to take action to keep cool, hence minimizing heat strain and by association, reducing the potential for health care utilization. However, effectiveness of this proof-of-concept early warning system remains to be evaluated in a robust trial design.

## Methods

### Study design

A convergent mixed-method study was designed with an aim to examine the association of the use of the Ethos system (the intervention) with heat awareness and knowledge and the uptake of cooling behaviours in a real world setting among the potential target users. Another purpose of this in-home proof-of-concept testing was to test the usability of the Ethos system.

### Study setting, participants, and duration

The study was conducted in Southeast Queensland (27.24°S 152.73°E) stretching from Noosa Heads in the north to Coolangatta in the south and west to Toowoomba. The region is Australia’s fastest growing urban area^64^ and has a rapidly ageing population^65^. It is also among one of the most at-risk regions in the world for high impact heatwaves^66,67^. It is characterised by a subtropical climate and experiences warm summers with average maximum temperatures of 29 °C often with high relative humidity and winter maximum temperatures averaging 20 °C^67^. The region experienced an El-Nino summer^63^ with record high average mean, maximum and minimum temperatures. Several sites experienced the warmest summer nights on record^68^.

**Fig. 4:**
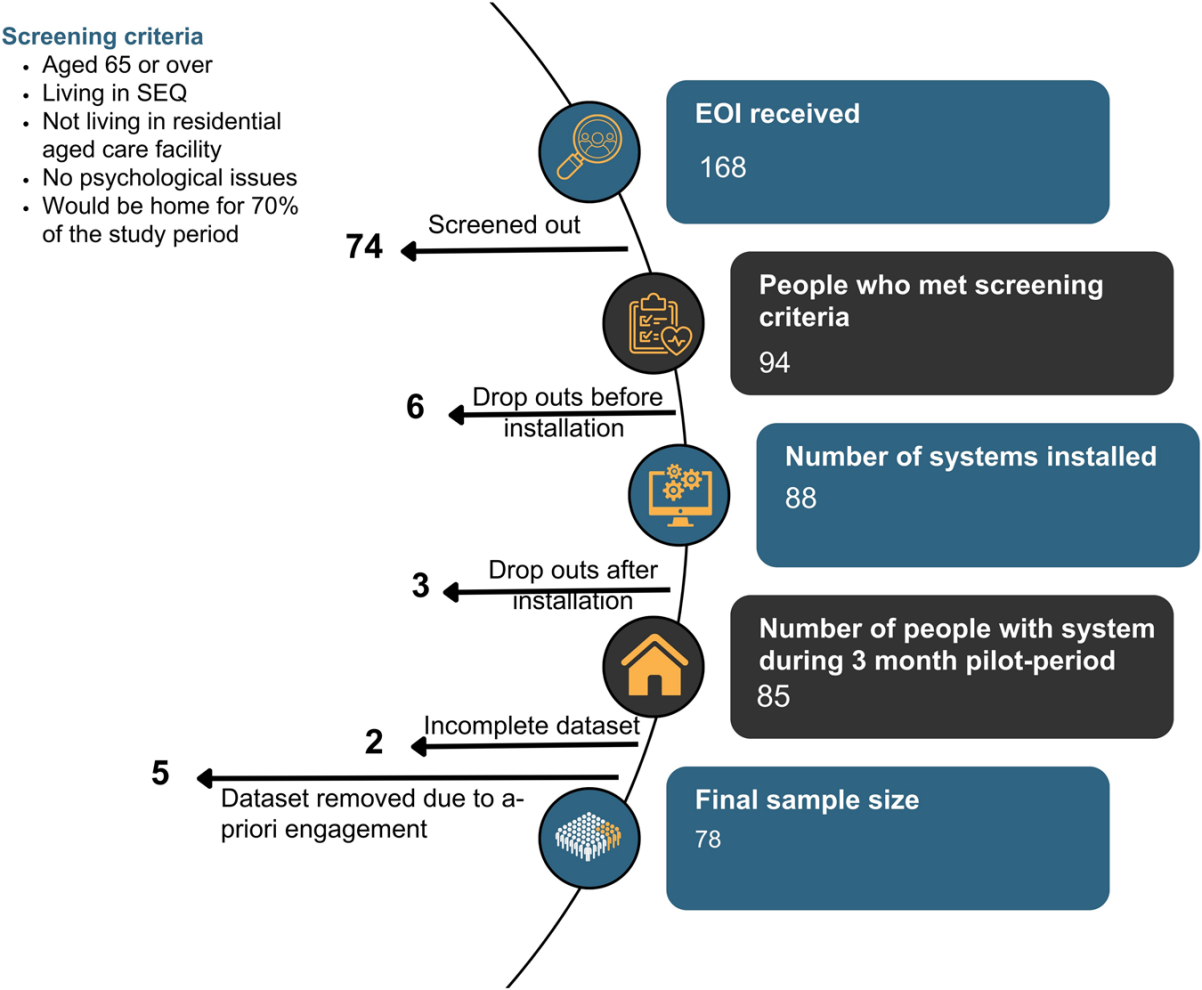
Participant recruitment process from the receipt of expressions of interest (EOI) to the final sample size. SEQ: Southeast Queensland. The diagram shows the number of participants left in each step of the recruitment process, from 168 EOIs received to 78 included in the final data analysis.

People were recruited for the study (Fig. 4) if they (i) were aged 65 or above, (ii) were living in Southeast Queensland, (iii) were not living in a residential aged care facility, (iv) were not suffering from psychological disorders, and (v) would be home for at least 70% of the study period. The participant screening questionnaire of the study is provided in Supplementary file. The intervention was deployed for the entire summer period, from 1^st^ December 2023 to 29^th^ February 2024.

### Sample size

A convenience sampling method was employed by the study accounting for the monetary/budget (resource) constraints^69^ which limited the number of Ethos systems that could be provided.

### Utilising a co-design participatory approach

Aligned with principles of consumer involvement in research^47^, the intervention was developed by the research team with active engagement of a formalised reference group comprising of people aged 65 or above or people who care for this age group (Ethics approval GUREF: 2023/022). They were consulted throughout all phases of the design process via online surveys, face to face or online design sessions, and World Cafe events with policy makers and researchers. They were also modestly remunerated for their time.

### Intervention: the Ethos System

**Fig. 5:**
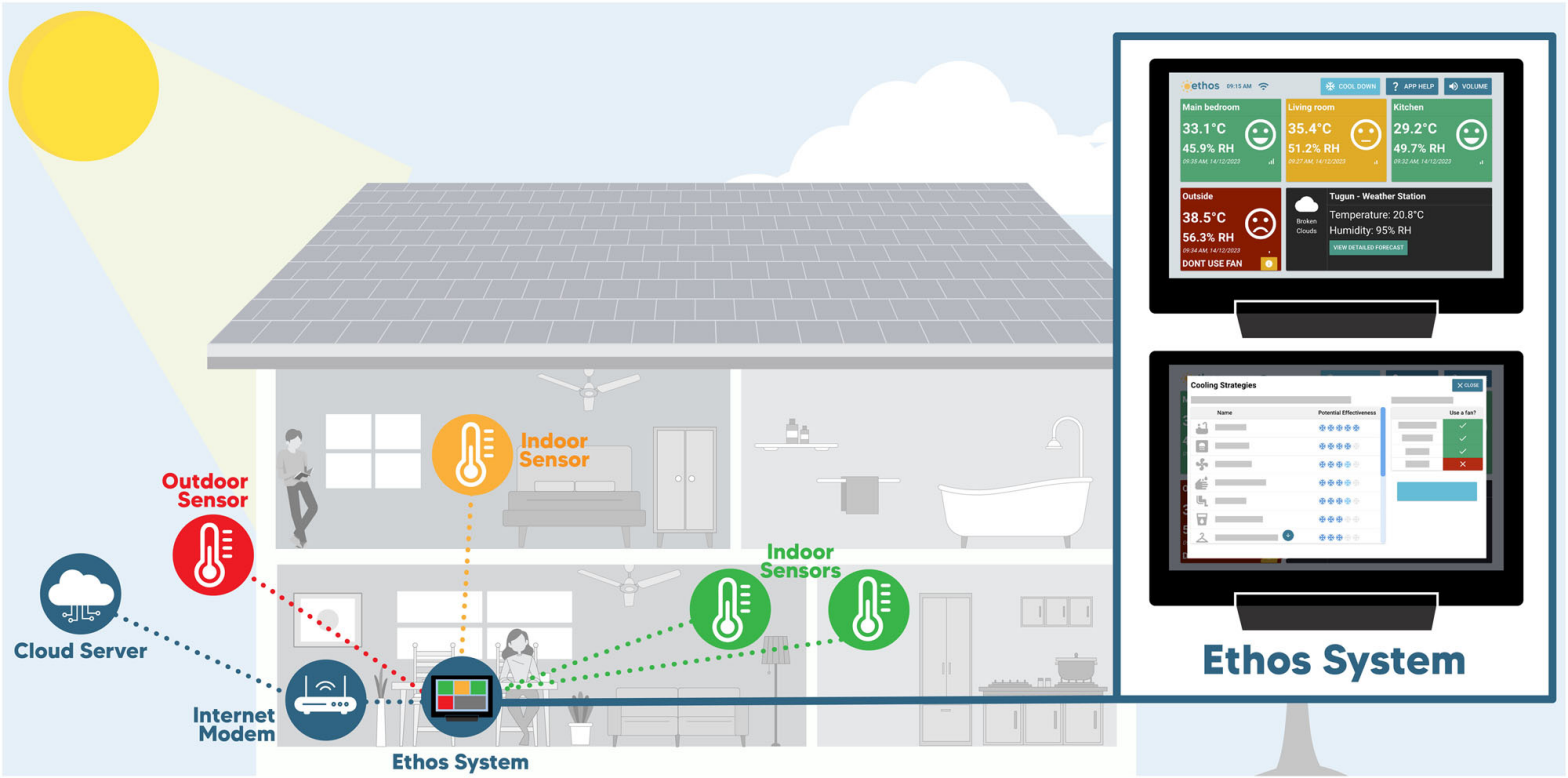
Schematic representation of the Ethos system with its key components and their positioning in the house of the participants. Three indoors sensors placed inside the different rooms (locations) in the house and the outdoor sensor can be clearly seen. A cloud server represents the secure research platform where data sent through the Wi-Fi router (internet modem) was stored. On the right-hand side of the figure, tablet screens as they appeared to the participants (users) are illustrated. Along with the data from the four sensors, participants had the option of referencing real-time weather and forecasted weather (provided by OpenWeatherMap) based on their postcode, through the Ethos system base station. The effective, accessible, and acceptable cooling measures are shown on the indicative screen at the bottom right section of this figure.

The Ethos system was designed to increase heat preparedness and resilience by improving heat awareness, improving individual heat health risk perception, and encouraging timely cooling action. It consists of three key physical component parts (Fig. 5): (i) four temperature-relative humidity sensors situated in four household locations: outside, in a living space, bedroom, and a third room most frequently occupied; (ii) a tablet interface for real-time data display, heat health risk prediction, alerting, and information for cooling; and (iii) a Wi-Fi router (internet modem), to send data to a secure research platform (cloud server). Weather forecasting functionality shows the current weather for the user, with a button that (when pressed) shows a pop-up window with the 5-day forecast. This was implemented using the OpenWeatherMap’s API, specifically the 5 day/3 h forecast endpoint, which shows weather information every 3 h over a 5-day timespan.

### Data Privacy

The Ethos system components communicate with server infrastructure through a dedicated cellular router provided to research participants. Each router employs Wi-Fi Protected Access 2 (WPA2) encryption protocol to secure the local wireless network. All data transmission between devices and the server occurs over Hypertext Transfer Protocol Secure (HTTPS), ensuring end-to-end encryption of data in transit. The server implements a CouchDB database with authentication requirements, restricting access to authorized users and devices only. Notably, no participant data is sent via WiFi, only sensor data (ID and room name, temperature, humidity) and alert logs.

**Fig. 6:**
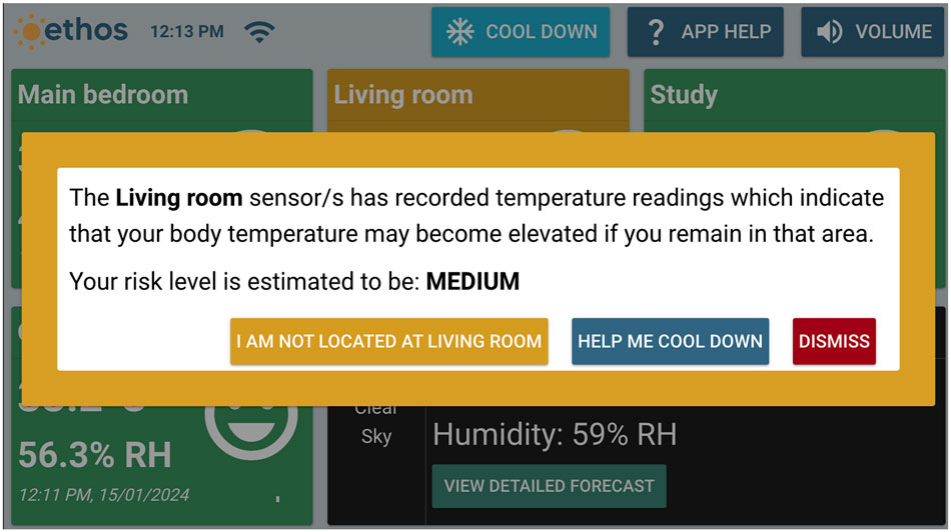
An example of a yellow alert issued by the Ethos system. As the temperature in the living room was high, the Ethos system issued a yellow alert. The participant can indicate if they were in the living room, ask for options to cool down, or dismiss the alert.

**Fig. 7:**
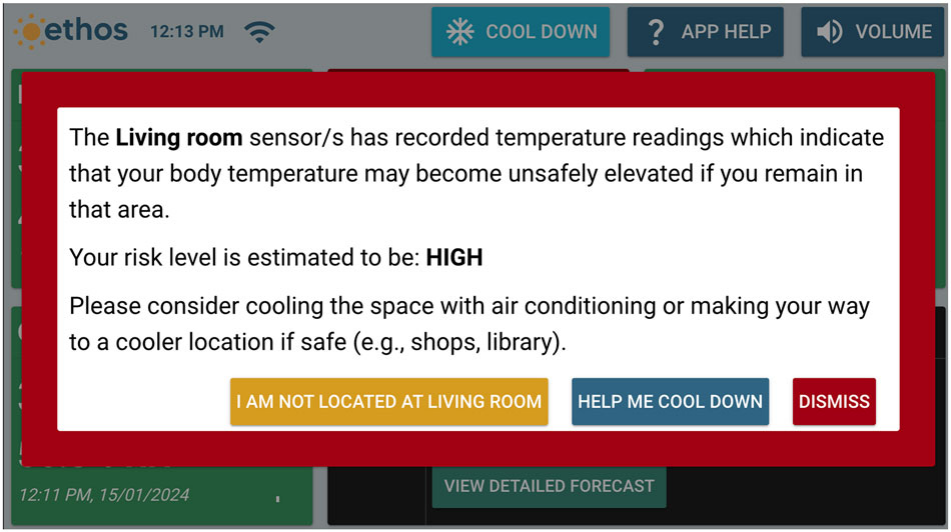
An example of a red alert issued by the Ethos system. As the temperature in the living room was very high, the Ethos system issued this red alert. The participant can indicate if they were in the living room, ask for options to cool down, or dismiss the alert.

The Ethos system is underpinned by an algorithm based on a heat-balance model. It utilised the multi-segment JOS-3 model^62^ to predict core body temperature of users in real-time based upon real-time ambient temperature and relative humidity of their respective house rooms, and the user’s age, sex, height, mass, physical activity ratio (PAR—assumed to be 1.6), air velocity (assumed to be 0.1 m/s), and insulation of clothing (CLO—assumed to be 0.45). The algorithm included three levels of risk stratification—normal, elevated, and hyperthermia (coinciding with green, yellow, and red screen indicators) on the basis of a change in projected core temperature. Each had a commensurate recommended action. The goal was for the projected individual’s core temperature to remain in the green (normal) zone. Based on the change in the core temperature projected within the forthcoming 9 h (assuming stable environmental conditions), the participant was alerted via colours as represented on the visual display of the system. Modest projected elevations in core temperature received a yellow alert, whereas high projected elevations in core temperature received a red alert (Fig. 6: yellow alert screen; Fig. 7: red alert screen). Alerts solely relied on recorded temperature and humidity data from the individual’s home, and their individual characteristics. The respective alerts would stay on the screen until the risk level came back to normal (green).

The system was set up for each user based upon their preference for alert (e.g., audio siren vs. voice assistant) and access to and acceptability of cooling strategies (e.g., presence of air-conditioning or a bathtub and willingness to use it). Commonly used cooling strategies were listed on the device^46^. Prior to implementation, participants were provided with educational resources regarding the operation of the system, as well as how to act on alerts and implement appropriate responses to issued warnings. This included disclaimers instructing participants to seek immediate medical attention if they were concerned for their health. Thereafter, participants were familiarised with the system, the menus, and provided with a printed “quick start guide” accompanying the device, which outlined basic functions of the system. The system included a feature to provide weather forecast details for up to 7 days based on participants nearest weather station.

### Data collection

This in-home proof-of-concept testing study consisted of three stages: (1) the pre-intervention stage, (2) the intervention stage, and (3) the post-intervention stage. The intervention was defined as the access to and use of the Ethos system from 1^st^ December 2023 to 29^th^ February 2024.

### Change in knowledge, awareness, preparedness, and cooling behaviours

**Fig. 8:**
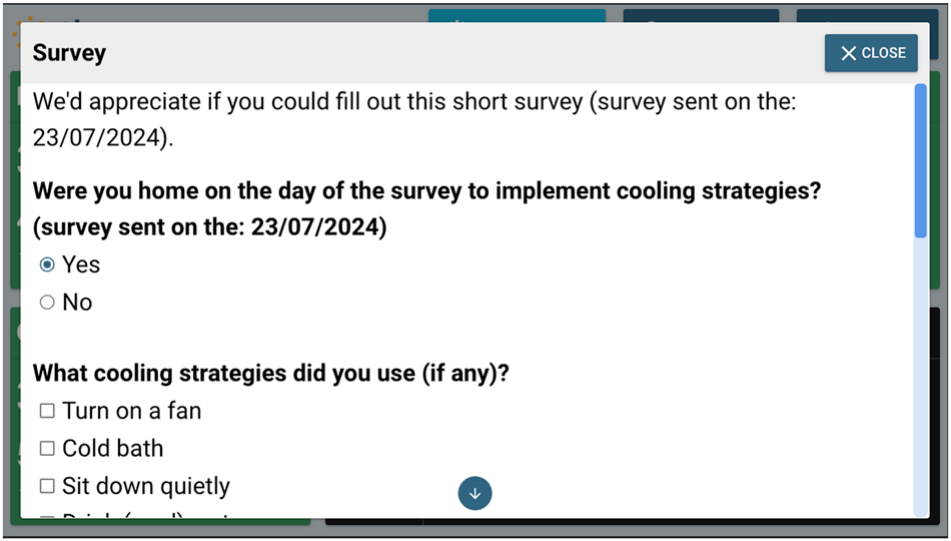
The first section of a mini pop-up survey issued by the Ethos system. When there was a yellow or red alert issued by the Ethos system during a day in a participant’s home, at 7 pm on that day, the Ethos system would send this pop-up survey. In this first section, the participant was asked if they were home to implement cooling strategies.

**Fig. 9:**
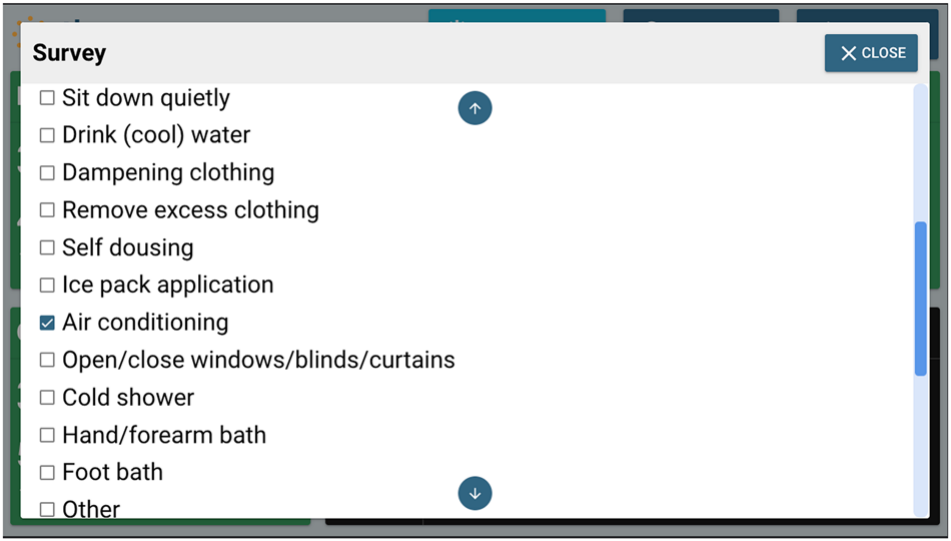
The second section of a mini pop-up survey issued by the Ethos system. When there was a yellow or red alert issued by the Ethos system during a day in a participant’s home, at 7 pm on that day, the Ethos system would send this pop-up survey. In this second section, the participant was asked which cooling strategies they used.

**Fig. 10:**
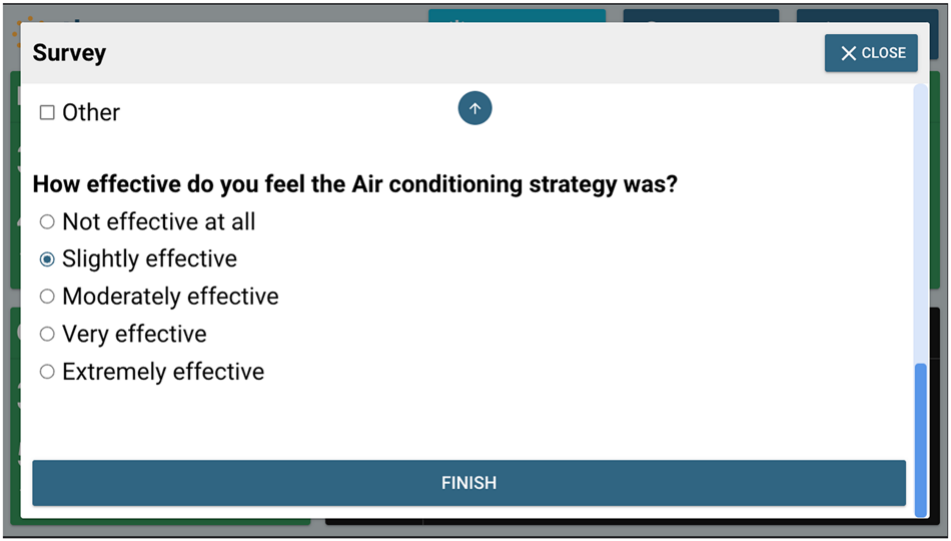
The third section of a mini pop-up survey issued by the Ethos system. When there was a yellow or red alert issued by the Ethos system during a day in a participant’s home, at 7 pm on that day, the Ethos system would send this pop-up survey. In this third section, the participant was asked if they found the cooling strategies they used effective.

Quantitative and qualitative data were collected at the pre- and post-intervention stages via an online or paper-based surveys (via REDCap electronic data capture tool hosted at Griffith University)^70,71^ to collect information on the participants’ heat-health risk knowledge, awareness, and preparedness, as well as personal and household cooling behaviours. Data was collected through mini pop-up surveys during the intervention stage (Figs. 8–10), which were administered on the Ethos system following a yellow or red alert.

The pre- and post-intervention surveys included a series of structured closed and open-ended questions (Supplementary file). The questions forming this part of the survey were designed and based on a review of the literature by the research team. The post-intervention survey included the same questions as the pre-intervention survey in addition to a few open-ended questions to elicit a better understanding on perspectives such as impact of the system, value to the user, and air conditioning usage patterns.

### Data on technology acceptance, system usability measures, and quality of life measurement

Standard survey tools and measures were utilised to measure technology acceptance, system usability, and quality of life among our participants.

Technology acceptance: The brief version of the Senior Technology Acceptance Model (STAM) with 14 items was included in the questionnaire to collect information on the acceptance of general technology among the participants^72^. This version includes four constructs relating to attitudinal beliefs, control beliefs, gerontechnology anxiety and health^72^. This scale has been previously validated for construct, internal consistency, and reliability^73^. The score for this scale ranges from 14 to 140, with a higher score meaning more acceptance of the technology in general.

Quality of life: The EQ-5D-5L scale was used for measuring the quality of life among the participants^74,75^ to gain a general understanding of the participant cohort involved in the study.

Usability of the Ethos system: Measurement of the usability of device/product is important for quantifying how well the users can interact with it^37^. System usability was measured using a positively worded validated and reliable System Usability Scale (SUS)^76,77^ following the field testing. Positively worded usability was utilised to reduce the cognitive load on the participants^43^. The scale was adapted to the Australian context and worded to probe specifically about the Ethos system, consisting of 10 positively framed statements. The scores were calculated and then interpreted using an adjective scale^38^. The final score of the scale ranges from 0 to 100, where higher scores mean high usability.

The study protocol was approved by the Human Research Ethics Committee of Griffith University (ethics application reference number: GUREF 2023/385). A written consent for participation was obtained from all the participants before the commencement of the study.

### Data analysis

The key research questions around improved heat preparedness among the users due to Ethos system and the intervention usability and acceptability were assessed using the quantitative and qualitative data.

The data analysis was carried out in SPSS (28.0), commencing with descriptive analysis to check for outliers, distribution and provide univariate measures for all the variable under study. The survey included categorical variables (for cooling behaviours, heat preparedness, heat knowledge, and heat-health risk perception), therefore McNemar test (for binomial categorical variables) and Marginal homogeneity test (for multinomial categorical variables) were employed to assess whether the marginal distributions of two related categorical variables were identical or not pre- and post the intervention (i.e., access to and use of the Ethos system). This non-parametric test is particularly suitable for comparing paired or matched data where each subject provides two related observations, often before and after an intervention^78^.

During the intervention stage, each day, when the Ethos system alerted following an elevated predicted core body temperature, the participant would see a pop-up survey on the messaging interface at 7 pm, asking if the person saw the alert and if they used any cooling measure and which cooling measures they used. We calculated the number of alerts (yellow or red) and the number of times when older adults used cooling measures after hearing an alert sent by the Ethos system.

Qualitative data analysis: Data were collected through a small set of open-ended responses included in the post-intervention survey (Q1: Did participation in the trial change your knowledge about how to respond to heat? Please explain; Q2: What was the impact of the Ethos system on you? Q3: What changed for you from having the Ethos system in your house? For the complete list of questions refer to the post-intervention survey in the Supplementary file). Qualitative analysis was guided by Braun and Clarke’s framework for thematic analysis^79^.

In the first phase of the qualitative data analysis, two researchers read through all the responses initially to familiarise themselves with the data. Following this, the initial coding was undertaken, where data was made more meaningful and pre-processed by removing stop words and punctuation and changing upper case to lower case. Next, data was read and re-read looking for major themes and categorising the responses into broader themes for the question. Following the manual analysis of the questions the pre-processed data was analysed using Leximancer to assist in themes generation. A mixed inductive and deductive approach was utilised throughout the process to unpack the findings supporting the quantitative data.

In reporting our qualitative findings, pseudonyms were used for participants with age identified^80^. While reporting the participants words, a pseudo name (representing the gender) with age was used in the following format (name, age in years).

## Supplementary information

Supplementary file

## Data Availability

The data used and/or analysed for this article are available from the corresponding author upon request. The data are not publicly available due to privacy or ethical restrictions.

## Code availability

All R codes used in the data analysis of this study are available from the corresponding author upon request.
