# Supplementary material for: A digital heat early warning system for older adults
Source: NPJ Digit Med. 2025 Feb 20;8:114. doi: 10.1038/s41746-025-01505-5 (PMC11840092; doi:10.1038/s41746-025-01505-5)
Supplement: Supplementary file 1 — Supplementary file [file 41746_2025_1505_MOESM1_ESM.pdf]

Supplementary file

A digital heat early warning system for older adults

Mehak Oberai, Zhiwei Xu, Aaron Bach, Connor Forbes, Ella Jackman, Fergus O'Connor, Isabella Ennever, Sebastian Binnewies, Steven Baker, & Shannon Rutherford

Contents of the supplementary file:

1. Supplementary Figure 1. Geographical locations of the participants' households.

2. Supplementary Table 1. Qualitative findings indicating improved heat preparedness.

3. Supplementary Table 2. Qualitative findings indicating change in awareness and knowledge.

4. Supplementary Table 3. Qualitative findings indicating improved heat adaptive behaviours leading to increased response capacity.

5. Supplementary Table 4. Qualitative findings regarding the usability of the system.

6. Pre-intervention survey questionnaire

7. Post-intervention survey questionnaire

8. Participant screening questionnaire of this in-home proof-of-concept testing study

9. STROBE checklist

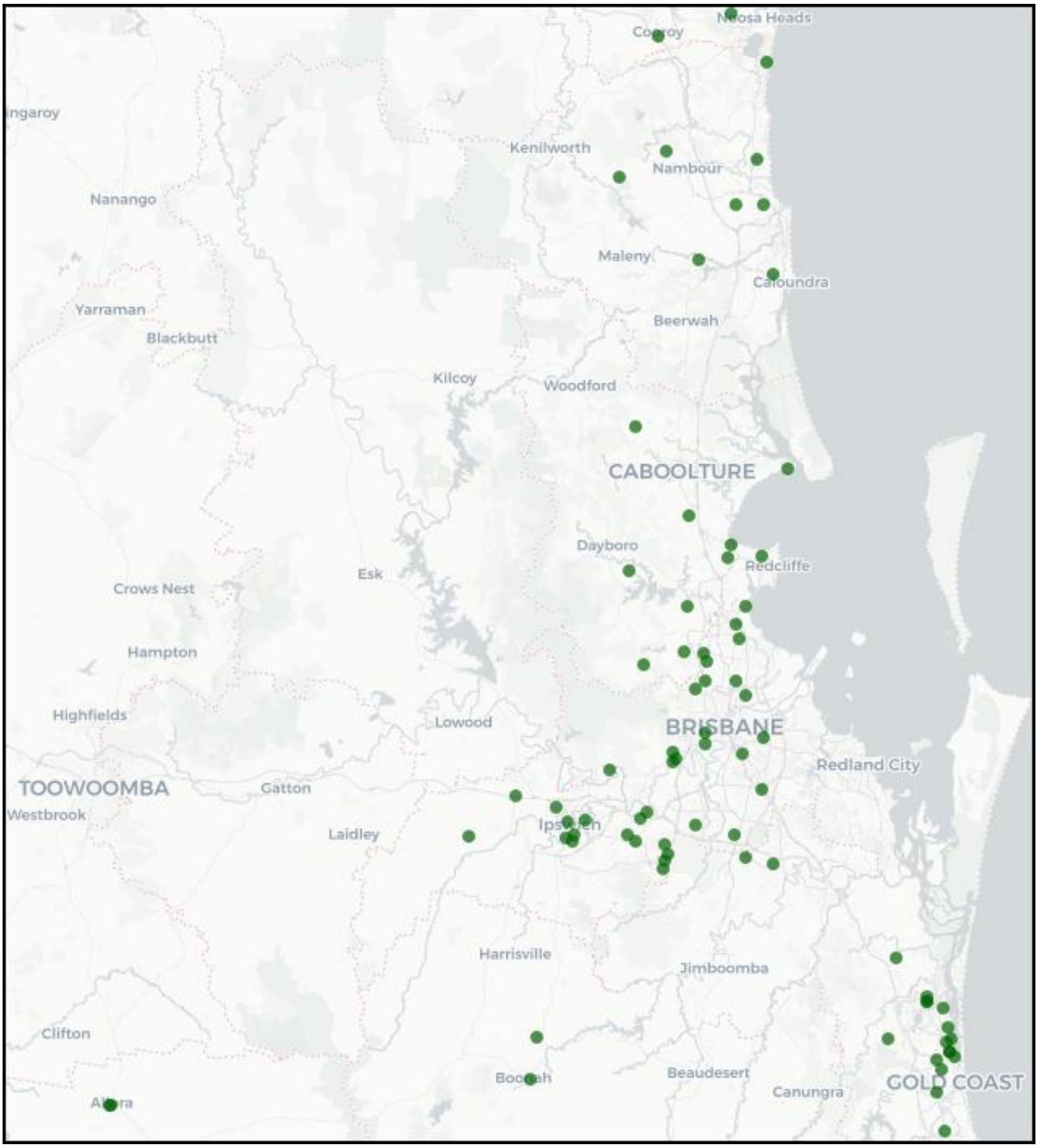

Supplementary Figure 1. Geographical locations of the 78 participants' households.

Supplementary Table 1. Qualitative findings indicating improved heat preparedness.

Theme	Examples supporting the theme
Improved personal and household preparedness	*"better prepared to handle the hot conditions" (Clara,70 yr)*<br><br>*"more prepared for planning my days"* (Arthur, 78 *yr*)<br><br>*"yes! Have developed a plan! If temperature is less than 30 deg relax. If 30 - 33 deg open up doors windows etc and use a fan, shed clothes. If greater than 33deg shut up house, set air conditioner to 27deg and turn on. If i am unsure where coolest part of house is, find the dog. Drink water!" (Alvin, 81 yr)*
Empowered participants	*"I developed a routine. When the temperature outside began to rise I would shut doors and windows; when the outside temperature was one degree or more less than the hottest indoor area I would open doors and windows. I also used the amber warnings as prompts to begin cooling strategies eg remove excess clothing." (Nick, 73 yr)*<br><br>*"We close off eastern facing windows as we go to bed & open western facing windows. Then we close off western facing rooms before 10.00am and open eastern facing curtains, blinds & windows after 1.00pm. Aircon is turned on in early morning if 30+° is forecast. All windows, curtains & blinds are closed and western rooms, not in use are closed. Aircon system was serviced for gas & efficiency because of Ethos" (Frank, 75 yr)*<br><br>*"It did change confidence in our response to high temperatures - we were able to make better informed decisions in relation to heatwaves in our house" (Louise, 76 yr)*
Preparedness for future	*"Convinced me I'm going to have to have air conditioning in at least one room, preferably a bedroom and the main room." (Nick, 73 yr)*<br><br>*"far better understanding of heat and the impact it has and what i need to do in the future" (Lara, 75 yr)*

Supplementary Table 2. Qualitative findings indicating change in awareness and knowledge.

Theme	Examples supporting the theme
Increased awareness and knowledge about heat-health risks	*"It quantified the threat posed by excessive heat and humidity on my health" (Mae, 68yr)*<br><br>*"far better understanding of heat and the impact it has and what i need to do in the future (Lara, 75 yr) "*<br><br>*"I had no idea of the implications heat had on my health and was in distress before the trial but now know I am in control" (Lena, 79yr)*
Improved personal perception of heat-health risks	*"… more conscious of the effect of going out in the noon day"*<br>*"Made me more aware of the effects of heat on older persons like myself." (Harvey, 69yr)*<br><br>*"I realised that I have a bodily reaction to the heat and the humidity at a much lower level than I thought" (Sadie, 72yr)*
Increased awareness and knowledge about temperature and humidity role in impacting the heat perception	*", it raised awareness of the confluence between temperature and humidity, and, in what situations, these two factors lead to taking specific actions" (Russell, 73yr)*<br><br>*"….told me the relative humidity which is not well enough publicized (in fact is relatively hidden) in the public weather systems." (Nick, 73yr)*
Increased knowledge and awareness of spatial and temporal surroundings	*"Felt better informed about the state of my climatic surroundings" (Harold, 78yr)*<br><br>*"I was more conscious of the temperature in my home and how to adjust my day accordingly. I didn't realise how hot it was inside my house." (Dorothy, 71yr)*<br><br>*"The most I got out of it was the regular display of the temperatures in each area of the house. I found that interesting and informative and it served to remind me of shading windows and sitting quietly and moving from room to room to the coolest spot in the house." (Florence, 69yr)*<br><br>*"I know which areas in the house are cooler and where to avoid when it gets hot." (*Della*, 75yr)*

Supplementary Table 3. Qualitative findings indicating improved heat adaptive behaviours leading to increased response capacity.

Theme	Examples supporting the theme
Increase in air-conditioning usage (more often, changed patterns, more consideration of efficiency, reassurance and confidence to use)	*“Probably used the air conditioning more and at an earlier time as the heat was commencing“ (Anna, 69yr)*<br><br>*“Probably use a fan or air conditioner earlier than I would have. Previously tended to 'put up with' heat & humidity longer than I should have” (Pearl, 71yr)*<br><br>*“More use of the air-conditioned. I also closed off the hottest rooms from the rest of the house” (Dorothy, 71yr)*<br><br>*“I had moral support when I needed to turn on the A/C and close up” (Emma, 100yr)*<br><br>*“It confirmed that what we have been doing previously (and did this summer) is based on sound health principles (Russell, 73yr)”*
Increased usage of fan use and in combination with air-conditioning	*“used fans more…… and used extraction fans in the bathroom to suck out hot air” (Kate, 70yr)*<br><br>*“Using fan and air conditioning at the same time. Never did this previously” (Gemma, 82yr)*<br><br>*“I found a combination approach useful. Fan A/C together with A/c at 26 worked well & saved money “ (Callum, 81yr)*
Awareness of more cooling options	*“I now know several solutions to help get cooler on hot days. I am now more aware of my environment and how best to cope during hot weather and especially heat waves.” (Della, 75yr)*<br><br>*“The trial suggested a number of cooling options which I hadn't previously thought of.” (Harvey, 69yr)*

Supplementary Table 4. Qualitative findings regarding the usability of the system

Theme	Examples supporting the theme
Liking towards the system design (interface, alerts, colours, simplicity, features)	*“I liked the size and the design. It was clear and the colours easily seen from a distance” (Emma, 100yr)*<br><br>*“The green and yellow (we never got a red!!) was easily noticeable even at a quick glance” (Russell,73yr)*<br><br>*”I liked the whole package: neat, easy to read & operate” (Esther, 86yr)*<br><br>*“I liked knowing the temperature and humidity levels in the different areas of my home. That way, I moved to the coolest area, or, turned on air conditioning, whichever was better at the time” (Daisy,76yr)*
Integration of the system into their life and decision making	*“It became part of the 'furniture'. A sentinel sitting in the corner, quietly waiting to activate” (Mae,68yr)*<br><br>*“A good friend and warner of heat outside” (Emma,100yr)*<br><br>*“I found myself checking the monitor every morning and preparing accordingly. My husband started checking it too and he kept asking me if we could keep it” (Kate,70yr)*<br><br>*“Impacted me to the extent that I would buy a device like this if it was generally available” (Henry, 69yr)*

Ethos In-home trials (IHT) Pre-trial Survey 2023-24

Thank you for participating in the Ethos In-home Trials this summer. In addition to having the device in your home, we are interested in your insights regarding heat health risk and awareness, as well as the behaviours you exhibit during hot weather. We would also like to assess your opinions on digital literacy over the course of this three-month period. To do so, we kindly request that you complete this pre-trial survey. This would take no more than 15 minutes to be completed.

While some questions may appear repetitive, it is because they are part of various standardized survey instruments we are utilizing. Your completion of the survey will be considered as consent for your participation. For more information about how your data will be stored please refer to research information pack (GU REF: 2023/385).

First Name ________________________________

Last Name ________________________________

The Bureau of Meteorology (BoM) defines heatwave as "three or more days of high maximum and minimum temperatures that are unusual for that location". For Queensland the temperature usually ranges from 37°C to 42°C for a heatwave period.
In this part of the survey, we will ask you some questions about what you think about heatwaves, and how you cope when it is hot.

Have you heard about heatwaves in the past?
- ◯ Yes
- ◯ No
- ◯ Not sure

Compared to the past, do you think there are more frequent heatwaves in Queensland?
- ◯ Yes
- ◯ No
- ◯ Don't know

How well informed do you think you are about heatwaves and their consequences for your health?
- ◯ Very well informed
- ◯ Fairly well informed
- ◯ Not very well informed
- ◯ Not at all informed

How concerned are you about the effects of heatwaves on you (personally)?
- ◯ Extremely concerned
- ◯ Very concerned
- ◯ Fairly concerned
- ◯ Bit concerned
- ◯ Not at all concerned

To what extent do you agree or disagree with the following?

	Strongly agree	Agree	Neither agree nor disagree	Disagree	Strongly disagree
Heatwave poses a risk to my health	◯	◯	◯	◯	◯
Heatwave poses a risk to the health of someone I know	◯	◯	◯	◯	◯

Do you think you are well prepared for heatwaves?
- ◯ Yes
- ◯ No
- ◯ Don't know

Please select all options that you usually use during hot weather to keep yourself cool.

- ☐ Drink plenty of water
- ☐ Take cool showers or splash yourself with cold water several times a day
- ☐ Go to an air-conditioned building in the local area (shopping mall, community centre, etc.)
- ☐ Go to a swimming pool
- ☐ Go to cool areas such as the beach or mountain or hilly areas
- ☐ Plan the day in a way that allows you to stay out of the heat
- ☐ Avoid strenuous activities
- ☐ Move to a cooler room in the house
- ☐ Check the weather forecast beforehand to be prepared for heatwave days
- ☐ Wear light coloured, loose fitting, lightweight clothes
- ☐ Remove excess clothing
- ☐ Water misting
- ☐ Dampening clothes
- ☐ Turn on the fans
- ☐ Turn on the air-conditioning
- ☐ Utilising windows/doors to improve indoor air conditions
- ☐ Keeping curtains closed on windows exposed to direct sunlight during the day
- ☐ Spend as much time as possible in shade
- ☐ Using ice packs
- ☐ Hand/forearm bath
- ☐ Foot bath
- ☐ Ask for help or help others to check on neighbours, family members or friends at least, twice on a day.
- ☐ Other
- ☐ None of the above.

If other is selected, please specify.

____

Do you change your usual cooling behaviour during a heatwave?

- ◯ Yes
- ◯ No

If yes, how regularly do you change your cooling behaviour?

- ◯ Always/every time during a heatwave
- ◯ Frequently
- ◯ Sometimes
- ◯ Rarely

If no, are there any reason(s) why you do not change your behaviour?

- ☐ Not necessary
- ☐ Not convenient
- ☐ Not possible (due to work, other commitments)
- ☐ Heat doesn't affect me
- ☐ Too expensive
- ☐ No access
- ☐ I already take precautions in the heat/ I know what to do
- ☐ Other
- ☐ Not sure

If other is selected, please specify.

____

How effective do you think the following actions are in protecting you from heat in the summer?

	Completely effective	Very effective	Somewhat effective	Slightly effective	Not at all effective	Do not use so cannot say
Drink plenty of water	○	○	○	○	○	○
Take cool showers or splash yourself with cold water several times a day	○	○	○	○	○	○
Go to an air-conditioned building in the local area (shopping mall, community centre,etc.)	○	○	○	○	○	○
Go to cool areas such as the beach or mountains or hilly areas	○	○	○	○	○	○
Avoid strenuous activities	○	○	○	○	○	○
Move to a cooler room in the house	○	○	○	○	○	○
Plan the day in a way that allows you to stay out of the heat	○	○	○	○	○	○
Check the weather forecast beforehand to be prepared for heatwave days	○	○	○	○	○	○
Utilising windows/doors to improve indoor air conditions	○	○	○	○	○	○
Keeping curtains closed on windows exposed to direct sunlight during the day	○	○	○	○	○	○
Wear light coloured, loose fitting, lightweight clothes	○	○	○	○	○	○
Remove excess clothing	○	○	○	○	○	○
Water misting	○	○	○	○	○	○
Dampening clothes	○	○	○	○	○	○
Turn on the fans	○	○	○	○	○	○
Turn on the air-conditioning	○	○	○	○	○	○
Using ice packs	○	○	○	○	○	○
Hand/forearm bath	○	○	○	○	○	○
Foot bath	○	○	○	○	○	○
Ask for help or help others to check on neighbours, family members or friends at least twice on a day.	○	○	○	○	○	○
Spend as much time as possible in shade	○	○	○	○	○	○

In the next part of the survey, we would like you to answer a few questions about technology.

Under each heading below, please rate each option from 1 (strongly disagree) to 10 (strongly agree) based on your feeling towards technology.

	1	2	3	4	5	6	7	8	9	10
Using technology enhances my effectiveness in daily activities.	○	○	○	○	○	○	○	○	○	○
I find technology is useful in my daily activities.	○	○	○	○	○	○	○	○	○	○
I like the idea of using technology.	○	○	○	○	○	○	○	○	○	○
I could be skilful at using technology.	○	○	○	○	○	○	○	○	○	○
I could complete a task using technology if there is someone to demonstrate how.	○	○	○	○	○	○	○	○	○	○
My financial status does not limit my activities in using technology.	○	○	○	○	○	○	○	○	○	○
When I want or need to use technology, it is accessible to me.	○	○	○	○	○	○	○	○	○	○
I feel apprehensive about using technology.	○	○	○	○	○	○	○	○	○	○
I hesitate to use technology for fear of making mistakes I cannot correct.	○	○	○	○	○	○	○	○	○	○

Please rate on the scale 1 (very poor) to 10 (very good).

	1	2	3	4	5	6	7	8	9	10
How is your general health condition?	○	○	○	○	○	○	○	○	○	○

Please rate on the scale 1 (not very easily) to 10 (very easily).

	1	2	3	4	5	6	7	8	9	10
How well are you able to concentrate?	○	○	○	○	○	○	○	○	○	○

Please rate on the scale 1 (Very unsatisfied) to 10 (very satisfied).

	1	2	3	4	5	6	7	8	9	10
How satisfied are you with your personal relationships?	○	○	○	○	○	○	○	○	○	○
How satisfied are you with the support received from friends and family?	○	○	○	○	○	○	○	○	○	○
How satisfied are you with your quality of life?	○	○	○	○	○	○	○	○	○	○

Under each heading below, please select one box that best describes your health today:

Thinking of "Mobility", please select one box that best describes your health today:	◯ I have no problems in walking about<br>◯ I have slight problems in walking about<br>◯ I have moderate problems in walking about<br>◯ I have severe problems in walking about<br>◯ I am unable to walk about
Thinking of "Self-care", please select one box that best describes your health today:	◯ I have no problems washing or dressing myself<br>◯ I have slight problems washing or dressing myself<br>◯ I have moderate problems washing or dressing myself<br>◯ I have severe problems washing or dressing myself<br>◯ I am unable to wash or dress myself
Thinking of "usual activities", please select one box that best describes your health today:	◯ I have no problems doing my usual activities<br>◯ I have slight problems doing my usual activities<br>◯ I have moderate problems doing my usual activities<br>◯ I have severe problems doing my usual activities<br>◯ I am unable to do my usual activities
Thinking of "pain/discomfort", please select one box that best describes your health today:	◯ I have no pain or discomfort<br>◯ I have slight pain or discomfort<br>◯ I have moderate pain or discomfort<br>◯ I have severe pain or discomfort<br>◯ I have extreme pain or discomfort
Thinking of "anxiety/depression", please select one box that best describes your health today:	◯ I am not anxious or depressed<br>◯ I am slightly anxious or depressed<br>◯ I am moderately anxious or depressed<br>◯ I am severely anxious or depressed<br>◯ I am extremely anxious or depressed
Overall, on a scale of 0-100 (where 0 is the worst health you can imagine, & 100 means the best health you can imagine), how is your health today? Please write in the given box.	__________________________

ETHOS post-trial survey 2023-24

Dear Valued Participant,

We extend our sincere gratitude for your active participation in the Ethos In-home Trials (trial) this summer. As the trial draws to a close, we wish to seek further your understanding of heat-health risks and awareness among older individuals, as well as your behaviours during hot weather. Additionally, we are seeking your inputs on how the Ethos system worked for you and its future utility.

To capture these insights comprehensively, we kindly request your participation in the post-trial survey. While some questions may seem repetitive, they are integral components of standardised survey instruments that enhance the depth of our analysis. Your thoughtful completion of the survey serves as consent for us to utilize the recorded data.

NOTE: COMPLETION OF THIS SURVEY WILL TAKE AROUND 10-20 MINUTES.

Best Regards,

Ethos team

First Name ________________________________

Last Name ________________________________

Age (in years) ________________________________

This section will collect information which would be stored anonymously and will be used by the research team to check for the sample representation.

What is your total personal income (weekly)?

- ◯ Negative/Nil income
- ◯ $1-$149
- ◯ $150-$299
- ◯ $300-$399
- ◯ $400-$499
- ◯ $500-$649
- ◯ $650-$799
- ◯ $800-$999
- ◯ $1,000-$1,249
- ◯ $1,250-$1,499
- ◯ $1,500-$1,749
- ◯ $1,750-$1,999
- ◯ $2,000-$2,999
- ◯ $3,000-$3,499
- ◯ $3,500 or more

What is your total household income (weekly)?

- ◯ Negative/Nil income
- ◯ $1-$149
- ◯ $150-$299
- ◯ $300-$399
- ◯ $400-$499
- ◯ $500-$649
- ◯ $650-$799
- ◯ $800-$999
- ◯ $1,000-$1,249
- ◯ $1,250-$1,499
- ◯ $1,500-$1,749
- ◯ $1,750-$1,999
- ◯ $2,000-$2,499
- ◯ $2,500-$2,999
- ◯ $3,000-$3,499
- ◯ $3,500-$3,999
- ◯ $4,000 or more

What is your highest education level?

- ◯ Postgraduate diploma/ certificate/degree
- ◯ Bachelor's degree
- ◯ Diploma or Advanced Diploma
- ◯ Certificate Level III or IV
- ◯ Secondary school education
- ◯ Primary school education
- ◯ None of the above

This section will collect information related to your housing.

You live in a (please select the most appropriate option)

- ◯ Unit or Apartment or flat (usually a complex of 2 or more storeys, with 1-3 bedrooms in each)
- ◯ Townhouse/Villa (3 or more units next to each other, with 1-4 bedrooms in each, divided by common walls)
- ◯ Duplex (usually 2 units, with 1-3 bedrooms in each, divided by a common wall, on 1 block of land)
- ◯ Dual occupancy (2 properties on 1 block of land)
- ◯ Free standing house (a house with 2 or more bedrooms on 1 block of land)
- ◯ Cluster house (several attached or separate homes, with 2-4 bedrooms in each, within a housing development)
- ◯ Seniors' unit (for people over the age of 55, usually in a complex of 1-2 storeys, with 1-2 bedrooms in each)
- ◯ Other

If other is selected, please specify. ______________________________

Your dwelling is made up of which material (please select the most appropriate options)

- ◯ Brick.
- ◯ Concrete.
- ◯ Mudbrick/rammed earth.
- ◯ Stone.
- ◯ Timber
- ◯ Other

If other is selected, please specify. ______________________________

Your dwelling is:

- ◯ Single storey
- ◯ Double storey
- ◯ Multiple storey
- ◯ Other

If other is selected, please specify. ______________________________

Number of bedrooms in your dwelling:

- ◯ 1
- ◯ 2
- ◯ 3
- ◯ 4
- ◯ 5 or more

Is your dwelling (please select the most appropriate option):

- ◯ Your own
- ◯ Rental
- ◯ Community housing/Public housing
- ◯ Other

If other is selected, please specify. ______________________________

Is your dwelling insulated?

- ◯ Yes
- ◯ No
- ◯ Don't know

If your dwelling is insulated, where is the insulation ?	☐ Roof<br>☐ Floor<br>☐ Walls<br>☐ Other
If other is selected, please specify.	__________________________________
If it is insulated, then what is the material the insulation is made of?	__________________________________

The Bureau of Meteorology (BoM) defines heatwave as "three or more days of high maximum and minimum temperatures that are unusual for that location". For Queensland the temperature usually ranges from 37°C to 42°C for a heatwave period.
In this part of the survey, we will ask you some questions about what you think about heatwaves, and how you cope when it is hot.

Have you heard about heatwaves in the past?	◯ Yes<br>◯ No<br>◯ Not sure
Compared to the past, do you think there are more frequent heatwaves in Queensland?	◯ Yes<br>◯ No<br>◯ Don't know
How well informed do you think you are about heatwaves and their consequences for your health?	◯ Very well informed<br>◯ Fairly well informed<br>◯ Not very well informed<br>◯ Not at all informed
How concerned are you about the effects of heatwaves on you (personally)?	◯ Extremely concerned<br>◯ Very concerned<br>◯ Fairly concerned<br>◯ Bit concerned<br>◯ Not at all concerned

To what extent do you agree or disagree with the following?

	Strongly agree	Agree	Neither agree nor disagree	Disagree	Strongly disagree
Heatwaves pose a risk to my health	◯	◯	◯	◯	◯
Heatwaves pose a risk to the health of someone I know	◯	◯	◯	◯	◯

Do you think you are well prepared for heatwaves?

◯ Yes
◯ No
◯ Don't know

Please select all options that you usually use during hot weather to keep yourself cool.

☐ Drink plenty of water
☐ Take cool showers or splash yourself with cold water several times a day
☐ Go to an air-conditioned building in the local area (shopping mall, community centre, etc.)
☐ Go to a swimming pool
☐ Go to cool areas such as the beach or mountain or hilly areas
☐ Plan the day in a way that allows you to stay out of the heat
☐ Avoid strenuous activities
☐ Move to a cooler room in the house
☐ Check the weather forecast beforehand to be prepared for heatwave days
☐ Wear light coloured, loose fitting, lightweight clothes
☐ Remove excess clothing
☐ Water misting
☐ Dampening clothes
☐ Turn on the fans
☐ Turn on the air-conditioning
☐ Utilising windows/doors to improve indoor air conditions
☐ Keeping curtains closed on windows exposed to direct sunlight during the day
☐ Spend as much time as possible in shade
☐ Using ice packs
☐ Hand/forearm bath
☐ Foot bath
☐ Ask for help or help others to check on neighbours, family members or friends at least, twice on a day.
☐ Other
☐ None of the above.

If other is selected, please specify.

____

Do you change your usual cooling behaviour during a heatwave?

◯ Yes
◯ No

If yes, how regularly do you change your cooling behaviour?

◯ Always/every time during a heatwave
◯ Frequently
◯ Sometimes
◯ Rarely

If no, are there any reason(s) why you do not change your behaviour?	☐ Not necessary<br>☐ Not convenient<br>☐ Not possible (due to work, other commitments)<br>☐ Heat doesn't affect me<br>☐ Too expensive<br>☐ No access<br>☐ I already take precautions in the heat/ I know what to do<br>☐ Other<br>☐ Not sure
If other is selected, please specify.	__________________________________

How effective do you think the following actions are in protecting you from heat in the summer?

	Completely effective	Very effective	Somewhat effective	Slightly effective	Not at all effective	Do not use so cannot say
Drink plenty of water	○	○	○	○	○	○
Take cool showers or splash yourself with cold water several times a day	○	○	○	○	○	○
Go to an air-conditioned building in the local area (shopping mall, community centre,etc.)	○	○	○	○	○	○
Go to cool areas such as the beach or mountains or hilly areas	○	○	○	○	○	○
Avoid strenuous activities	○	○	○	○	○	○
Move to a cooler room in the house	○	○	○	○	○	○
Plan the day in a way that allows you to stay out of the heat	○	○	○	○	○	○
Check the weather forecast beforehand to be prepared for heatwave days	○	○	○	○	○	○
Utilising windows/doors to improve indoor air conditions	○	○	○	○	○	○
Keeping curtains closed on windows exposed to direct sunlight during the day	○	○	○	○	○	○
Wear light coloured, loose fitting, lightweight clothes	○	○	○	○	○	○
Remove excess clothing	○	○	○	○	○	○
Water misting	○	○	○	○	○	○
Dampening clothes	○	○	○	○	○	○
Turn on the fans	○	○	○	○	○	○
Turn on the air-conditioning	○	○	○	○	○	○
Using ice packs	○	○	○	○	○	○
Hand/forearm bath	○	○	○	○	○	○
Foot bath	○	○	○	○	○	○
Ask for help or help others to check on neighbours, family members or friends at least twice on a day.	○	○	○	○	○	○
Spend as much time as possible in shade	○	○	○	○	○	○

In the next part of the survey, we would like you to answer a few questions about technology.

Under each heading below, please rate each option from 1 (strongly disagree) to 10 (strongly agree) based on your feeling towards technology.

	1	2	3	4	5	6	7	8	9	10
Using technology enhances my effectiveness in daily activities.	○	○	○	○	○	○	○	○	○	○
I find technology is useful in my daily activities.	○	○	○	○	○	○	○	○	○	○
I like the idea of using technology.	○	○	○	○	○	○	○	○	○	○
I could be skilful at using technology.	○	○	○	○	○	○	○	○	○	○
I could complete a task using technology if there is someone to demonstrate how.	○	○	○	○	○	○	○	○	○	○
My financial status does not limit my activities in using technology.	○	○	○	○	○	○	○	○	○	○
When I want or need to use technology, it is accessible to me.	○	○	○	○	○	○	○	○	○	○
I feel apprehensive about using technology.	○	○	○	○	○	○	○	○	○	○
I hesitate to use technology for fear of making mistakes I cannot correct.	○	○	○	○	○	○	○	○	○	○

Please rate on the scale 1 (very poor) to 10 (very good).

	1	2	3	4	5	6	7	8	9	10
How is your general health condition?	○	○	○	○	○	○	○	○	○	○

Please rate on the scale 1 (not very easily) to 10 (very easily).

	1	2	3	4	5	6	7	8	9	10
How well are you able to concentrate?	○	○	○	○	○	○	○	○	○	○

Please rate on the scale 1 (Very unsatisfied) to 10 (very satisfied).

	1	2	3	4	5	6	7	8	9	10
How satisfied are you with your personal relationships?	○	○	○	○	○	○	○	○	○	○
How satisfied are you with the support received from friends and family?	○	○	○	○	○	○	○	○	○	○
How satisfied are you with your quality of life?	○	○	○	○	○	○	○	○	○	○

**Under each heading below, please select one box that best describes your health today:**	
Thinking of "Mobility", please select one box that best describes your health today:	◯ I have no problems in walking about<br>◯ I have slight problems in walking about<br>◯ I have moderate problems in walking about<br>◯ I have severe problems in walking about<br>◯ I am unable to walk about
Thinking of "Self-care", please select one box that best describes your health today:	◯ I have no problems washing or dressing myself<br>◯ I have slight problems washing or dressing myself<br>◯ I have moderate problems washing or dressing myself<br>◯ I have severe problems washing or dressing myself<br>◯ I am unable to wash or dress myself
Thinking of "usual activities", please select one box that best describes your health today:	◯ I have no problems doing my usual activities<br>◯ I have slight problems doing my usual activities<br>◯ I have moderate problems doing my usual activities<br>◯ I have severe problems doing my usual activities<br>◯ I am unable to do my usual activities
Thinking of "pain/discomfort", please select one box that best describes your health today:	◯ I have no pain or discomfort<br>◯ I have slight pain or discomfort<br>◯ I have moderate pain or discomfort<br>◯ I have severe pain or discomfort<br>◯ I have extreme pain or discomfort
Thinking of "anxiety/depression", please select one box that best describes your health today:	◯ I am not anxious or depressed<br>◯ I am slightly anxious or depressed<br>◯ I am moderately anxious or depressed<br>◯ I am severely anxious or depressed<br>◯ I am extremely anxious or depressed
Overall, on a scale of 0-100 (where 0 is the worst health you can imagine, & 100 means the best health you can imagine), how is your health today? Please write in the given box.	______________________________

The next section includes questions relating to the usability of the Ethos system.

To what extent you agree or disagree with the following statements:

	Strongly disagree	Disagree	Neither agree nor disagree	Agree	Strongly agree
I think that I would like to use the Ethos system frequently	○	○	○	○	○
I found the Ethos system to be simple	○	○	○	○	○
I thought the Ethos system was easy to use	○	○	○	○	○
I think that I could use the Ethos system without the support of a technical person	○	○	○	○	○
I found the various functions in the Ethos system were well integrated	○	○	○	○	○
I thought there was a lot of consistency in the Ethos system	○	○	○	○	○
I imagine that most people would learn to use the Ethos system very quickly	○	○	○	○	○
I found the Ethos system very intuitive	○	○	○	○	○
I felt very confident using the Ethos system	○	○	○	○	○
I could use the Ethos system without having to learn anything new	○	○	○	○	○

This section includes questions for your feedback on general aspects of the in-home trial and impact that Ethos system has made in your lives over the past two to three months.

Did participation in the trial change your knowledge about how to respond to heat? Please explain.	__________________________________
What was the impact of the Ethos system on you?	__________________________________
What changed for you from having the Ethos system in your house?	__________________________________
Did participation in the trial change your confidence to respond to heat? - please explain.	__________________________________
Can you share if there were any cooling options, that you started to use because you participated in our study?	__________________________________
Can you share if there were any cooling options, that you used more often because you participated in our study?	__________________________________
Did your air-conditioner usage change because of Ethos system?	◯ Yes-used more<br>◯ Yes-used less<br>◯ No-it remained the same
Were there factors that prevented you from using the Ethos system? Please explain.	__________________________________
What did you like most about the Ethos interface -eg. size, design, information, or any other feature?	__________________________________
What did you like least about the Ethos interface - eg. size, design, information, or any other feature?	__________________________________
Would you be interested in using a feature that sent an alert to a carer/family member?	◯ Yes<br>◯ No
Please explain your choice above	__________________________________

What would you prefer?	◯ an app for your smartphone, similar to a weather app (i.e.., BOM) which shows your personal heat health risk and sends alerts to your phone (similar to the Ethos system)<br>◯ base station like the one you had during this study for monitoring personalised heat health risks<br>◯ None of the above<br>◯ Something else
Please explain your choice above.	____
How much would you be willing to pay for a system with base station?	◯ $100<br>◯ $150<br>◯ $200<br>◯ more than $200
How do you think the current Ethos system can be improved to cater to the needs of older people when dealing with heat? Please provide as many suggestions as you wish as we will consider this feedback to refine our system for the second trial next summer.	____
Would you like to be involved in future trials of this system?	◯ Yes<br>◯ No<br>◯ Not sure
We will be sending a short report to all the participants about their data such as temperature and humidity averages during the summer season, number of alerts that were triggered by our system, etc. Are you interested in receiving the report?	◯ Yes<br>◯ No
Any other suggestions to the team about the system.	____

Ethos IHT Screening Questionnaire (GUREF: 2023/385)

Please complete the screening questionnaire below. This is important as this will help us in determining your eligibiltiy to participate in our study. In case of any questions please feel free to send us an email at ethos@griffith.edu.au or give us a call on (07) 555 279 03.

Regards!

Ethos Team

Griffith University

First name	________________________
Last Name	________________________
You live in (Please select the most suitable option):	◯ Ipswich<br>◯ Moreton Bay<br>◯ Brisbane<br>◯ Sunshine Coast<br>◯ Gold Coast<br>◯ Logan<br>◯ Scenic Rim<br>◯ Somerset<br>◯ Lockyer valley<br>◯ Redland<br>◯ Toowoomba
Home occupancy between December 1st, 2023 and February 29th, 2024 (please select the most suitable option)	◯ Not going on holidays<br>◯ Planned holidays away from your home
If you have planned holidays during Dec 2023 to Feb 2024, please specify the time you'll be away from your home.	◯ Less than a month<br>◯ More than a month
What is your mobile phone provider? (e.g., Telstra, Optus, Vodafone, etc.)	________________________
Do you have an air conditioner at your home?	◯ Yes<br>◯ No
You have an air conditioner (please tick all that apply):	☐ In your bedroom<br>☐ In your living room<br>☐ In your kitchen<br>☐ In most/all of your home (e.g., ducted)<br>☐ Another place e.g., study, where you spend most of your daytime hours at home
Please specify where you spend most of the daytime hours at home, if last option was selected previously.	______________________________

Generally, across these spaces, how often would you use it?	◯ Never<br>◯ Rarely<br>◯ Only when it's hot<br>◯ Only at night to sleep<br>◯ All throughout summer when at home<br>◯ Other
If other is selected, please explain.	__________________________________
Please select the most appropriate option, about your living situation:	◯ Living alone<br>◯ Living with your partner only<br>◯ Living with your family/friends<br>◯ Other, please specify
How many family members/friends you live with?	____________________________
If other option is selected above, please specify:	____________________________

Do you have problems with the following?

	Yes	No
Mobility? e.g., walking, using stairs, balance:	◯	◯
Agility? e.g., bending, reaching up, kneeling down	◯	◯
Dexterity? e.g., getting dressed, writing, using tools	◯	◯
Physical Exertion? e.g., lifting, carrying, running:	◯	◯
Communication? e.g., speech, hearing	◯	◯
Vision? e.g., visual impairment, colour blindness, tunnel vision	◯	◯

If YES to any of the above, please give full details (e.g., extent of impairment, how you manage, support needs):	__________________________________

Do you have any of the following medical conditions?

	Yes	No
Cardiac Disorder? e.g., heart failure, high blood pressure.	◯	◯

Neurological Disorder? e.g., epilepsy, multiple sclerosis, Parkinson's disease, dementia.	◯	◯
Respiratory Disorder? e.g., asthma, chronic obstructive pulmonary disorder (COPD).	◯	◯
Kidney Disorder? e.g., chronic kidney disease (CKD).	◯	◯
Endocrine Disorder? e.g., diabetes, hypothyroidism.	◯	◯
Other Chronic Disorders? e.g., cancer.	◯	◯

If YES to any of the above please give details including an indication of date and duration etc (e.g., when condition developed, severity, management, current state): ________________________________

Are you a current or recent (within 6 months) smoker?
◯ Yes
◯ No

Are you currently prescribed or taking any of the following prescription or over-the-counter medications or classes of medications?

	Yes	No	Don't know
Anticholinergics (medications to reduce urine frequency, e.g., Enablex, Ditropan, Vesicare, Atropt)	◯	◯	◯
Anti-adrenergics and Beta-blockers (Medications for high blood pressure, e.g., atenolol (e.g., Noten), metoprolol (e.g., Betaloc), nebivolol (e.g., Nebilet), oxyprenolol (e.g., Corbeton))	◯	◯	◯
Antihistamines (Medications to treat allergies, e.g., Benadryl, Chlor-Trimeton, Zyrtec, Claritin, Allegra)	◯	◯	◯
Vasoconstrictors (Pressor medications or medications to relieve lower blood pressure, e.g., Naphcon Forte)	◯	◯	◯

Diuretics (Fluid medications, e.g., amiloride (Kaluril) and spironolactone (Aldactone))	○	○	○
Antiarrhythmics (medications for irregular heartbeat, e.g., Digoxin, Lanoxin, Sigmaxin)	○	○	○
Anticoagulants (also known as blood thinners, e.g., Warfarin, Coumadin, Marevan)	○	○	○
Antiepileptics/anticonvulsant (e.g., Diamox, Tegretol, Rivotril, Keppra)	○	○	○
Biguanides (High blood sugar medications or medications to treat Diabetes, e.g., Diabex, APX- Metformin, Metex XR, Glucobete, Malarone, Metformin GH)	○	○	○
Angiotensin-converting enzyme (ACE) inhibitors (Medications for high blood pressure, e.g., Perindopril, Ramipril, Lisniopril, Enalapril, Quinapril)	○	○	○
Antipsychotics or neuroleptics (typical and atypical) (Medications for mood, e.g., aripiprazole (e.g. Abilify), Clozapine (e.g. Clozaril), olanzapine (e.g. Zypine))	○	○	○
Antidepressants (e.g, sertraline, citalopram, escitalopram)	○	○	○
Non-steroidal anti-inflammatory drugs (NSAIDS) (e.g., Panadol, Nurofen , Voltaren , Aspirin, Ibuprofen, Naproxen)	○	○	○

If you are not sure about the above classes of medications or if you take medications other than those in the list above, please give the details below. __________________________________

Anything else that you would like to add which you think would help us in decision making and is of relevance to our research project? __________________________________

STROBE Statement—checklist of items that should be included in reports of observational studies

	Item No.	Recommendation	Page No.	Relevant text from manuscript
**Title and abstract**	1	(*a*) Indicate the study's design with a commonly used term in the title or the abstract		
		(*b*) Provide in the abstract an informative and balanced summary of what was done and what was found	2	
**Introduction**				
Background/rationale	2	Explain the scientific background and rationale for the investigation being reported	2,3,4	
Objectives	3	State specific objectives, including any prespecified hypotheses	4	
**Methods**				
Study design	4	Present key elements of study design early in the paper	13	
Setting	5	Describe the setting, locations, and relevant dates, including periods of recruitment, exposure, follow-up, and data collection	13,14,15	
Participants	6	(*a*) *Cohort study*—Give the eligibility criteria, and the sources and methods of selection of participants. Describe methods of follow-up<br>*Case-control study*—Give the eligibility criteria, and the sources and methods of case ascertainment and control selection. Give the rationale for the choice of cases and controls<br>*Cross-sectional study*—Give the eligibility criteria, and the sources and methods of selection of participants	13,14,15	
		(*b*) *Cohort study*—For matched studies, give matching criteria and number of exposed and unexposed<br>*Case-control study*—For matched studies, give matching criteria and the number of controls per case		
Variables	7	Clearly define all outcomes, exposures, predictors, potential confounders, and effect modifiers. Give diagnostic criteria, if applicable	15,16	
Data sources/ measurement	8*	For each variable of interest, give sources of data and details of methods of assessment (measurement). Describe comparability of assessment methods if there is more than one group	13,14,15,16	
Bias	9	Describe any efforts to address potential sources of bias	4	
Study size	10	Explain how the study size was arrived at	4	

Continued on next page

Quantitative variables	11	Explain how quantitative variables were handled in the analyses. If applicable, describe which groupings were chosen and why	16,17
Statistical methods	12	(*a*) Describe all statistical methods, including those used to control for confounding	16,17
		(*b*) Describe any methods used to examine subgroups and interactions	NA
		(*c*) Explain how missing data were addressed	NA
		(*d*) *Cohort study*—If applicable, explain how loss to follow-up was addressed<br>*Case-control study*—If applicable, explain how matching of cases and controls was addressed<br>*Cross-sectional study*—If applicable, describe analytical methods taking account of sampling strategy	NA
		(*e*) Describe any sensitivity analyses	NA
**Results**			
Participants	13*	(a) Report numbers of individuals at each stage of study—eg numbers potentially eligible, examined for eligibility, confirmed eligible, included in the study, completing follow-up, and analysed	4
		(b) Give reasons for non-participation at each stage	4
		(c) Consider use of a flow diagram	13
Descriptive data	14*	(a) Give characteristics of study participants (eg demographic, clinical, social) and information on exposures and potential confounders	4,5
		(b) Indicate number of participants with missing data for each variable of interest	NA
		(c) *Cohort study*—Summarise follow-up time (eg, average and total amount)	NA
Outcome data	15*	*Cohort study*—Report numbers of outcome events or summary measures over time	
		Case-control study—Report numbers in each exposure category, or summary measures of exposure	
		Cross-sectional study—Report numbers of outcome events or summary measures	
Main results	16	(*a*) Give unadjusted estimates and, if applicable, confounder-adjusted estimates and their precision (eg, 95% confidence interval). Make clear which confounders were adjusted for and why they were included	5,6,7,8
		(*b*) Report category boundaries when continuous variables were categorized	5,6,7
		(*c*) If relevant, consider translating estimates of relative risk into absolute risk for a meaningful time period	

Continued on next page

Other analyses	17	Report other analyses done—eg analyses of subgroups and interactions, and sensitivity analyses	NA
**Discussion**			
Key results	18	Summarise key results with reference to study objectives	8,9,10
Limitations	19	Discuss limitations of the study, taking into account sources of potential bias or imprecision. Discuss both direction and magnitude of any potential bias	11,12
Interpretation	20	Give a cautious overall interpretation of results considering objectives, limitations, multiplicity of analyses, results from similar studies, and other relevant evidence	8,9,10,11,12
Generalisability	21	Discuss the generalisability (external validity) of the study results	8,9,10,11,12
**Other information**			
Funding	22	Give the source of funding and the role of the funders for the present study and, if applicable, for the original study on which the present article is based	18

*Give information separately for cases and controls in case-control studies and, if applicable, for exposed and unexposed groups in cohort and cross-sectional studies.

Note: An Explanation and Elaboration article discusses each checklist item and gives methodological background and published examples of transparent reporting. The STROBE checklist is best used in conjunction with this article (freely available on the Web sites of PLoS Medicine at http://www.plosmedicine.org/, Annals of Internal Medicine at http://www.annals.org/, and Epidemiology at http://www.epidem.com/). Information on the STROBE Initiative is available at www.strobe-statement.org.
